# Knowledge-based Fragment Binding Prediction

**DOI:** 10.1371/journal.pcbi.1003589

**Published:** 2014-04-24

**Authors:** Grace W. Tang, Russ B. Altman

**Affiliations:** 1 Department of Bioengineering, Stanford University, Stanford, California, United States of America; 2 Department of Genetics, Stanford University, Stanford, California, United States of America; Bar Ilan University, Israel

## Abstract

Target-based drug discovery must assess many drug-like compounds for potential activity. Focusing on low-molecular-weight compounds (fragments) can dramatically reduce the chemical search space. However, approaches for determining protein-fragment interactions have limitations. Experimental assays are time-consuming, expensive, and not always applicable. At the same time, computational approaches using physics-based methods have limited accuracy. With increasing high-resolution structural data for protein-ligand complexes, there is now an opportunity for data-driven approaches to fragment binding prediction. We present FragFEATURE, a machine learning approach to predict small molecule fragments preferred by a target protein structure. We first create a knowledge base of protein structural environments annotated with the small molecule substructures they bind. These substructures have low-molecular weight and serve as a proxy for fragments. FragFEATURE then compares the structural environments within a target protein to those in the knowledge base to retrieve statistically preferred fragments. It merges information across diverse ligands with shared substructures to generate predictions. Our results demonstrate FragFEATURE's ability to rediscover fragments corresponding to the ligand bound with 74% precision and 82% recall on average. For many protein targets, it identifies high scoring fragments that are substructures of known inhibitors. FragFEATURE thus predicts fragments that can serve as inputs to fragment-based drug design or serve as refinement criteria for creating target-specific compound libraries for experimental or computational screening.

## Author Summary

In drug discovery, the goal is to identify new compounds to alter the behavior of a protein implicated in disease. With the very large number of small molecules to test, researchers have increasingly studied fragments (compounds with a small number of atoms) because there are fewer possibilities to evaluate and they can be used to identify larger compounds. Computational tools can efficiently assess if a fragment will bind a protein target of interest. Given the large number of structures available for protein-small molecule complexes, we present in this study a data-driven computational method for fragment binding prediction called FragFEATURE. FragFEATURE predicts fragments preferred by a protein structure using a knowledge base of all previously observed protein-fragment interactions. Comparison to previous observations enables it to determine if a query structure is likely to bind particular fragments. For numerous protein structures bound to small molecules, FragFEATURE predicted fragments matching the bound entity. For multiple proteins, it also predicted fragments matching drugs known to inhibit the proteins. These fragments can therefore lead us to promising drug-like compounds to study further using computational tools or experimental resources.

## Introduction

In recent years, the productivity of pharmaceutical research and development has declined [1], [2]. Although the Human Genome Project and associated disease studies have increased the number of potential protein targets [3], development of effective new drugs has been slow. The key steps in drug discovery involve hit identification and subsequent optimization of these leads into drug candidates. While the latter can be the more difficult task, hit identification is far from solved. In hit identification, a fundamental challenge is the prohibitive number of compounds to assess for bioactivity against a protein target; small molecule databases like ZINC [4] and PubChem [5] have grown rapidly as new synthetic capabilities emerge [6]. Moreover, databases with computationally enumerated molecules like GDB-17 [7] contain billions of compounds. Indeed, the universe of molecules up to 30 atoms in size may exceed 10^60^ members, though not all are synthetically feasible or drug-like [8]. Experimental high-throughput screening and computational virtual screening are the main approaches for identifying drug leads. However, experimental screening requires significant investment in equipment and screens on the order of a million compounds, just a sliver of “chemical space” [9]. Computational methods, of which docking algorithms are dominant, have much higher throughput but limited predictive accuracy [10].

Given the difficulty in thoroughly exploring the chemical space of drug-like molecules, efforts to study fragments have emerged. Fragments in this context refer to low-molecular-weight small molecules usually 120–250 Daltons in weight [11], [12] that combine to form larger molecules. Fragments have higher hit rates compared to large, complex drug-like molecules because they are less likely to possess suboptimal interactions or physical clashes with the protein [13]. A fragment library can provide a more compact and tractable basis set for chemical space than standard small molecule libraries [11]. Fragment-based drug discovery has also had recent success [14], [15], identifying favorable fragments that are “grown” or “linked” to form larger drug-like compounds that bind a protein target with high affinity. This process also improves the specificity, as fragments alone are less specific than larger molecules. Initial identification of fragments that bind to a protein target, however, is non-trivial.

Fragments tend to bind in the millimolar to micromolar range and require sensitive experimental screening techniques, including protein crystallography [16], [17], nuclear magnetic resonance (NMR) spectroscopy [18], [19], and surface plasmon resonance [20]. Characteristics of the fragments and protein targets, such as fragment solubility and protein stability, affect the applicability of these techniques [12]. There are also experimental difficulties such as assay sensitivity, experimental timescale, and equipment and infrastructure cost. Computational approaches are free from many of these concerns and can achieve much higher throughput but have limited predictive capabilities. Fragment docking (like ligand docking) is difficult. For uncertain reasons, the best-performing algorithms vary with the protein target studied, making algorithm selection difficult [10]. Additionally, binding and non-binding fragments may be indistinguishable by the scoring functions used, because fragments bind with weak affinity and a small inaccuracy in the predicted binding energy of 1.4 kcal/mol is a ten-fold difference in affinity [10]. Scoring functions also struggle to distinguish correct and incorrect fragment poses [21]. Other physics-based methods like SILCS immerse the protein target in a solution of fragments that compete for binding over the course of molecular dynamics simulations [22]. There are assumptions and approximations about molecular interactions built into these methods, limiting their ability to recapitulate natural interactions. For example, scoring functions may not include the energetic cost of ligand desolvation and force fields may not model atomic polarizability. These methods also require user input of a fragment test set, potentially introducing testing bias. If inhibitor knowledge for a protein target is available, user input of a fragment test set derived from these compounds can be desirable. However, it is not a systematic approach and risks limiting the scope of discovery by focusing on particular types/classes of fragments.

However, the growing database of structural data for protein-small molecule complexes provides an opportunity for empirical data-driven approaches to fragment binding prediction [23]. Empirical methods need not make assumptions regarding the forces governing molecular interactions and have the potential to predict rather than evaluate fragments. Wang *et. al*. took a ligand fragment-centric view and calculated the residue preferences of different fragments [24]. Chan *et. al.* took a protein residue-centric view and determined the fragment preferences of the side chains of Asp, Glu, Arg, and His [25]. These studies have some limitations. First, they captured protein information using residues, ignoring any effects the biochemical and biophysical environment around the residues might have had on fragment binding. Additionally, both focused on frequencies of interactions, marginalizing less common residue-fragment interactions that are real but observed less frequently within the PDB [26]. Lastly, they showed strong residue-fragment interaction patterns but did not extend this to automated fragment prediction given a target protein structure.

Here, we describe FragFEATURE, a novel fragment binding predictor that overcomes many of the limitations of existing *in silico* fragment binding predictors/evaluators. Using information from the Protein Data Bank, we created a knowledge base linking local protein structural environments to the small molecule fragments they bind. Given structural environments from a target protein, FragFEATURE compares them to the knowledge base to find similar structural environments and identify statistically preferred fragments. For a variety of protein-ligand complexes, we demonstrate the method's ability to predict fragments that are substructures of the bound ligands. We also present six case studies (three in main text and three in supporting information) of fragment predictions corresponding to known inhibitors. These fragment predictions can help identify promising compounds from compound libraries. Such compounds have potential to form favorable interactions with the protein target due to the presence of the fragment but require additional evaluation as other moieties in the compounds may prevent binding. Predicted fragments are also starting points for fragment-based drug design. Furthermore, knowledge of fragments favored by a protein pocket could give insight to lead optimization by suggesting favorable chemical groups. FragFEATURE thus powerfully leverages empirical knowledge of protein-fragment interactions to predict fragments for a target protein structure.

## Results

### Knowledge base of protein-fragment associations

**Figure 1:**
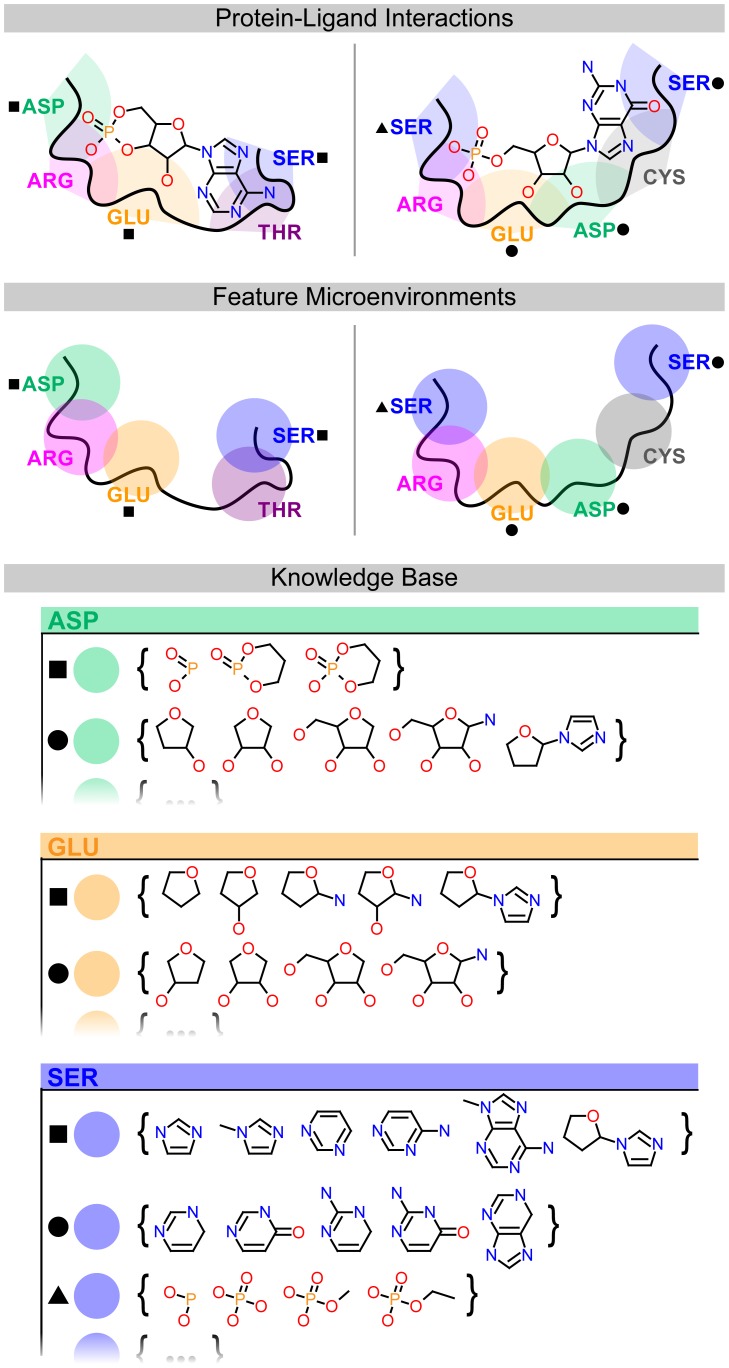
Knowledge base of protein microenvironments linked to ligand fragments. For each protein-ligand complex from the PDB, we identify residue atoms interacting with the ligand and note the ligand atoms proximal to them (semi-transparent shaded regions) (top). Next, the FEATURE microenvironments of the residue atoms are calculated (semi-transparent circles) (center). We then map ligand atoms to their pre-computed fragment lists and link them to their proximal microenvironments to form the knowledge base (bottom).

The knowledge base is the underlying source of information for fragment predictions. We mined 34,000 protein-ligand complexes from the PDB (01/01/2013 snapshot) to create a knowledge base of local protein structural environments (microenvironments) annotated with the small molecule substructures (fragments) they bind (Figure 1). In the knowledge base, there are 1.7 million microenvironments and 225 thousand unique fragments with 250 million connections between microenvironments and individual fragments.

To capture protein information, we used FEATURE [27] to represent protein microenvironments–the physicochemical properties of a local region of protein structure computed and represented as a vector of numbers (FEATURE is summarized in Figure S1). FEATURE divides the local environment around a point of interest into six concentric shells and evaluates 80 physicochemical properties within each, including atom type, hydrophobicity, secondary structure, etc. This converts a local structural environment (microenvironment) into a vector representation of length (6×80 = ) 480. There are 23 microenvironment types in the knowledge base centered on residue side chains or the protein backbone. The prevalence of each microenvironment type within binding pockets varies significantly, indicating they are not uniformly involved in ligand binding (Figure S2).

To capture fragment information, we divided 16,000 PDB ligands into overlapping substructures ranging from three to thirteen heavy atoms in size (Figure S3). These fragments correspond to molecules from the PubChem Compound database and have a PubChem identifier number for referencing. Fragmentation of the PDB ligands produced 225,000 unique fragments with fragments of different sizes having different prevalence (Figure S4A). The number of microenvironments linked to each fragment also varies greatly, indicating different amounts of information regarding the microenvironments that bind each fragment (Figure S4B). Some fragments are substructures of many and/or common ligands and thus associated with many microenvironments. Others are substructures of few and/or uncommon ligands and thus associated with few microenvironments.

### FragFEATURE robustly predicts fragments of validation ligands

**Figure 2:**
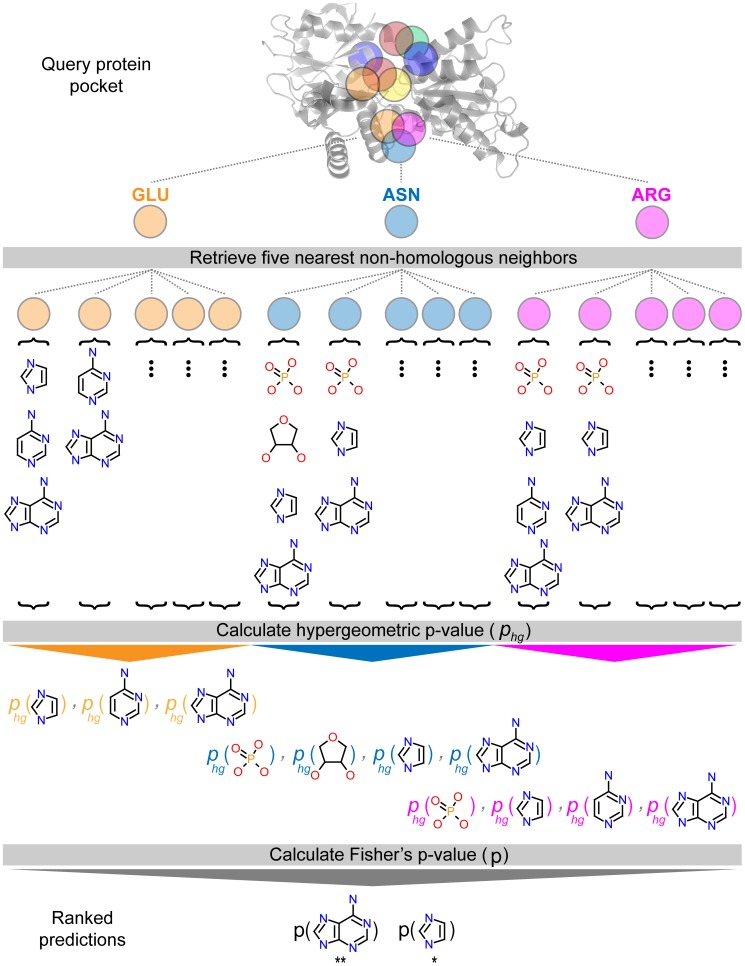
FragFEATURE predicts fragments for a protein pocket of interest. Given a pocket of interest as a series of microenvironments (semi-transparent circles), we compare each microenvironment to knowledge base microenvironments of the same type to retrieve the five most similar non-homologous neighbors. Each neighbor has a list of bound fragments for which a hypergeometric p-value is determined. For spatially proximal microenvironments (orange, blue, and magenta circles), we combine fragment hypergeometric p-values for shared fragments to generate Fisher's p-values. Denoted with an asterisk are statistically significant fragments with p-value(**)<p-value(*).

FragFEATURE predicts fragments preferred by the microenvironments of a protein pocket (Figure 2). It uses the assumption that a microenvironment will bind the same fragments as its “nearest neighbor” (most similar) microenvironments (Text S1) (Figure S5). It returns fragment predictions for sets of locally proximal microenvironments that represent sub-regions of a protein pocket (Figure S6). Each fragment prediction only uses information from non-homologous proteins to prevent trivial predictions. As the knowledge base contains all available protein-ligand interactions, FragFEATURE dynamically filters for homology. It selects nearest neighbor microenvironments/proteins such that they share less than 50% sequence identity to the query protein and to each other. The effective size of the knowledge base thus changes with the structure under evaluation.

We validated FragFEATURE on four nucleotide and four non-nucleotide ligands and found strong ability to predict fragments matching the ligand bound. The nucleotides included adenine (ADE), adenosine-5′-diphosphate (ADP), flavin-adenine dinucleotide (FAD), and nicotinamide-adenine dinucleotide (NAD). The non-nucleotides included thiamin (VIB), thiamine diphosphate (TPP), pyridoxal-5′-phosphate (PLP), and triclosan (TCL). These compounds tested FragFEATURE on ligands of different frequency (Table S1), flexible ligands, and various chemical moieties in different chemical contexts. We treat each ligand as a set of chemical moieties (Figure S7) to identify those parts of a ligand that the predicted fragments recapture.

**Figure 3:**
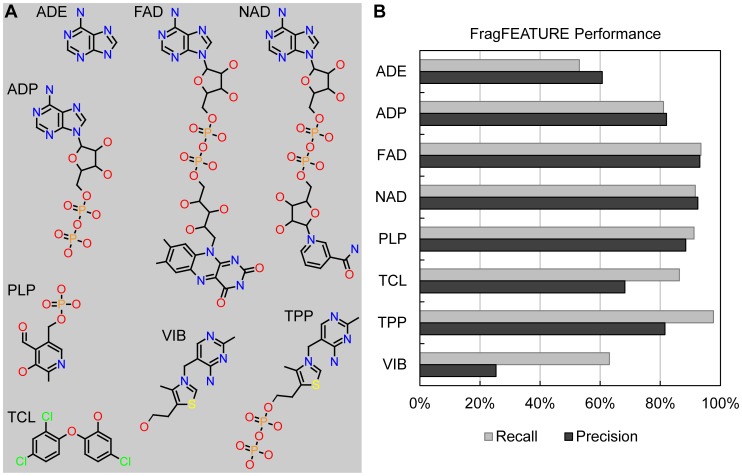
FragFEATURE performance on the validation ligands. A) Chemical structure (heavy atoms) of each validation ligand. B) FragFEATURE recall and precision on each validation ligand.

FragFEATURE predicted fragments for 9,392 ligand-binding pockets (Table S2) and we assessed performance using recall and precision. Recall measures the fraction of bound ligand moieties recaptured by the predicted fragments. Precision measures the fraction of fragment predictions that are correct. Predictions are labeled (1) correct, (2) incorrect, or (3) no information (i.e. no ligand information is available to assess a fragment's validity) (Figure S8). For the validation ligand-binding pockets, FragFEATURE achieves an average recall of 82% and precision of 74% (Figure 3). For each test structure, the most significant correct fragment prediction uses information from nearest neighbor proteins sharing low sequence identity with the test structure (∼30%) (Figure S9). In many cases, nearest neighbor proteins fail to align to the test structure.

For the previous analysis, we used observed ligand-binding pockets. In other applications of FragFEATURE, the location and extent of the ligand-binding pockets may be unknown. In those cases, both pocket definition and conformation may alter the microenvironments used by FragFEATURE to make predictions. To determine the sensitivity to non-ideal microenvironments, we tested computationally predicted pockets (fPocket [28]) from both ligand-bound and ligand-free structures (Figure S10). We analyzed the largest pocket of each protein chain as most small molecules bind proteins in their largest pocket [29] or largest predicted pocket [30]. For ligand-bound structures, we observe a slight increase in recall (+3%) and decrease in precision (−8%) across 9,121 predicted pockets (Table S2) (Text S2) (Figure S11). For ligand-free structures, we observe a stronger decrease in both recall (−18%) and precision (−12%) across 5,155 predicted pockets (Table S2) (Text S3) (Figure S11).

### FragFEATURE predicts fragments of inhibitors

We investigated the potential utility of FragFEATURE fragment predictions for drug discovery. In the following case studies, FragFEATURE rediscovered fragments of inhibitors for the analyzed protein targets. These “drug-relevant fragments” can inform different research approaches including virtual screening, high-throughput experimental screening, or fragment-based drug design.

### Exotoxin A

**Figure 4:**
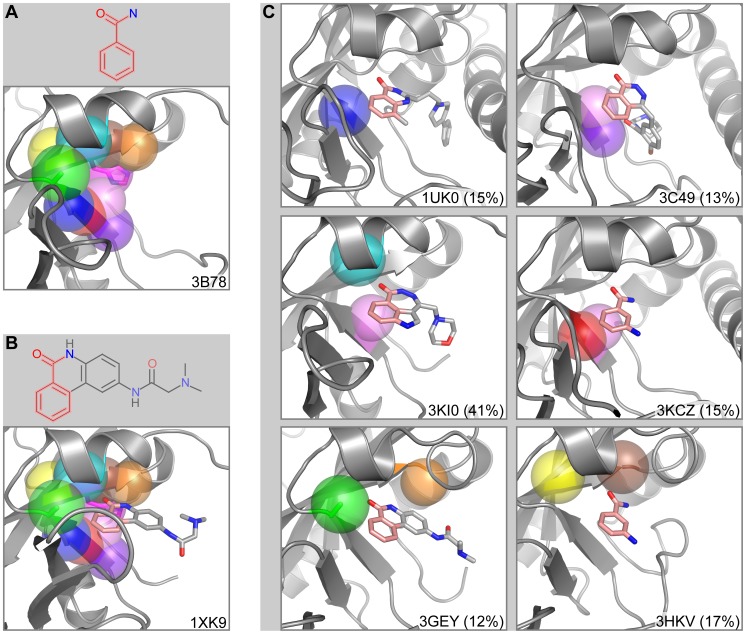
Fragment prediction and validation for exotoxin A. A) Fragment 2331 (benzamide) and the microenvironments from the query exotoxin A structure associated with the fragment prediction. B) PDB ligand P34 and an alternate structure of exotoxin A bound to P34. The benzamide substructure of P34 is in pink. C) Example nearest neighbor microenvironments. The benzamide substructure of the bound ligands is in pink. The percent sequence identity between each knowledge base structure and exotoxin A is in parentheses. 1UK0, 3C49, 3KCZ, 3GEY, and 3HKV are members of the poly [ADP-ribose] polymerase superfamily while 3KI0 is cholix toxin. Proteins are shown in cartoon representation with microenvironments as semi-transparent spheres. Microenvironment color scheme is arbitrary but consistent between panels. Side chains corresponding to microenvironments are shown in stick representation. Ligands are also drawn in stick representation.

*Pseudomonas aeruginosa* is an opportunistic bacterium that infects immunocompromised patients [31]. Key to its potency is exotoxin A, an ADP-ribosyltransferase that triggers cell death through inactivation of eukaryotic ribosomal elongation factor 2. We studied the catalytic domain of exotoxin A bound to its endogenous NAD ligand (PDB ID: 3B78 [32]). FragFEATURE predicts fragment 2331 (benzamide) with a p-value of 5.1×10^−29^ for the microenvironments proximal to the nicotinamide moiety of NAD (Figure 4A). Ten microenvironments centered on the side chain and/or backbone of residues Tyr439, His440, Gly441, Tyr470, Ile471, Ala472, and Ala478 contribute to the prediction. Their nearest neighbors bind seventeen rare, benzamide-containing PDB ligands including 09L, 0RU, 0RY, 18N, 3AB, 4AN, 78P, BZC, DHQ, FRM, FRQ, G9D, G9G, G9H, G9L, KU8, and P34 (Table S3) (Figure 4C). Interestingly, one benzamide-containing ligand, P34, is an exotoxin A inhibitor (K_i_: 140 nM) and is available in the PDB bound to exotoxin A (PDB ID: 1XK9 [33]). This structure shows the benzamide-preferring microenvironments to be in contact with the benzamide substructure of P34 (Figure 4B), validating it as a relevant drug fragment. The nearest neighbor microenvironments show high local structural similarity to the query and to each other (Figure 4C). They are predominantly from the poly [ADP-ribose] polymerase (PARP) superfamily (Tables S4 and S5), whose catalytic activity of ADP-ribosylation uses NAD as the substrate [34]. As the fragment prediction is in the active site of exotoxin A, FragFEATURE unsurprisingly retrieves fragment information from proteins with a similar catalytic activity. However, pairwise sequence identity between the proteins is low, averaging 15% using the PDB's jFATCAT-flexible server [35], [36] or 21% using the DaliLite server [37] (Table S6). FragFEATURE thus identifies locally conserved fragment binding motifs in proteins that have globally diverged. This example highlights FragFEATURE's ability to aggregate information across diverse protein structures and ligands to detect fragment enrichment. We observe similar capabilities in our predictions for the related cholix toxin from *Vibrio cholera* (Text S4) (Table S3).

### Death-associated protein kinase 1 (DAPK1)

**Figure 5:**
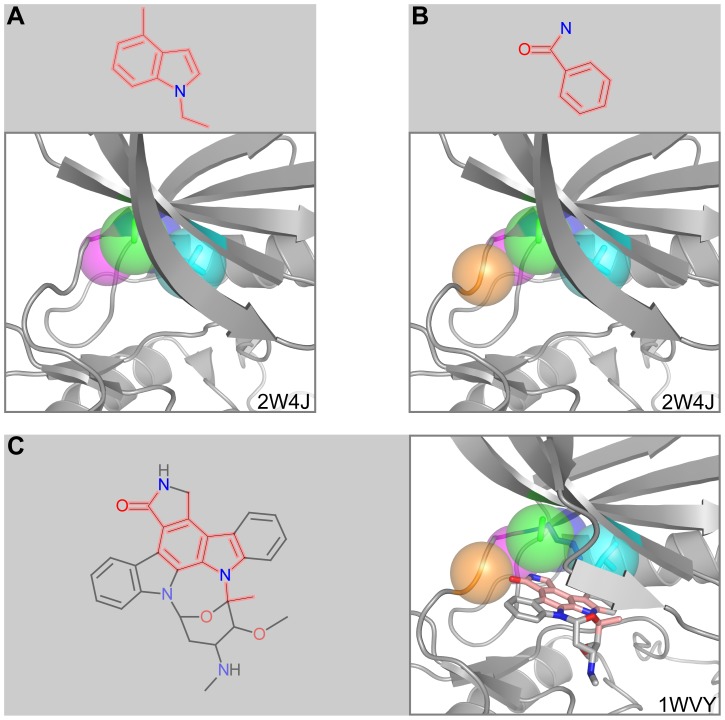
Fragment prediction and validation for DAPK1. A) Fragment 13509097 and the microenvironments from the query DAPK1 structure associated with the fragment prediction. B) Fragment 2331 (benzamide) and the microenvironments from the query DAPK1 structure associated with the fragment prediction. C) PDB ligand STU and an alternate structure of DAPK1 bound to STU. Fragment 13509097 and 2331 substructures of STU are in pink. Proteins are shown in cartoon representation with microenvironments as semi-transparent spheres. Microenvironment color scheme is arbitrary but consistent between panels. Side chains corresponding to microenvironments are shown in stick representation. Ligands are also drawn in stick representation.

DAPK1 is a serine/threonine kinase involved in regulating cell survival, apoptosis, and autophagy [38]. We analyzed the kinase domain in complex with ADP (PDB ID: 2W4J [39]) and found four microenvironments binding the adenine moiety of ADP to prefer fragment 13509097 with a p-value of 1.1×10^−9^ (Figure 5A). These microenvironments from residues Val27, Ala40, Leu93, and Glu94 when combined with a fifth from Val96 prefer fragment 2331 (benzamide) with a p-value of 1.0×10^−8^ (Figure 5B). The protein structures that FragFEATURE uses fragment information from consists of serine/threonine kinases, tyrosine kinases, or dual specificity kinases from *Homo sapiens* (Table S7). Their average sequence identity to DAPK1 is 24% (jFATCAT) or 27% (DaliLite) (Table S8). While FragFEATURE also predicts benzamide for exotoxin A, the benzamide here originates from different PDB ligands, primarily STU (staurosporine) but also 0CE, 609, KSA, and SKE (Table S9). Importantly, FragFEATURE provides the information supporting a fragment prediction, elucidating differences between seemingly identical fragment predictions. Staurosporine, the main ligand contributing to both predictions, is an inhibitor of DAPK1 (K_D_: 1.4 nM). A crystal structure of staurosporine-bound DAPK1 (PDBID: 1WVY [40]) reveals the predicted fragments to be overlapping and in close proximity to the microenvironments preferring them (Figure 5C). These inhibitor fragments demonstrate non-independent predictions, where the fragments are co-occurring and overlapping substructures of a bioactive compound. This suggests the larger molecule formed by the aggregation of these fragments may be important for bioactivity. We also observe similar non-independent predictions with Abl tyrosine kinase (Text S5) (Figure S12).

### Atypical protein kinase C (aPKC)

**Figure 6:**
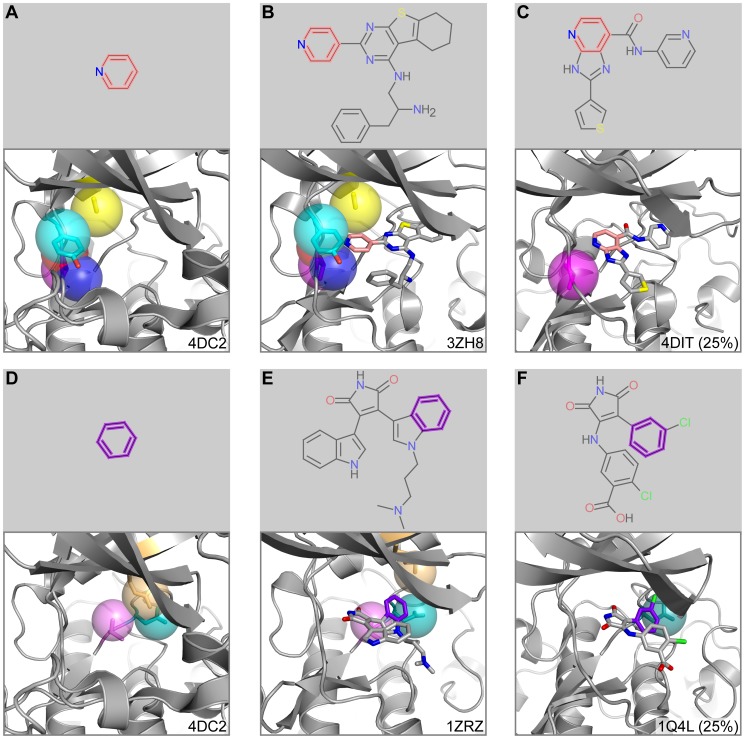
Fragment prediction and validation for aPKC. A) Fragment 1049 and the microenvironments from the query aPKC structure associated with the fragment prediction. B) PDB ligand C58 and an alternate structure of aPKC bound to C58. Fragment 1049 substructure of C58 is in pink. C) Example nearest neighbor microenvironment from GSK3β. Fragment 1049 of the bound PDB ligand 0KD is in pink. The percent sequence identity between GSK3β and aPKC is in parentheses. D) Fragment 241 and the microenvironments from the query aPKC structure associated with the fragment prediction. E) PDB ligand BI1 and an alternate structure of aPKC bound to BI1. Fragment 241 substructure of BI1 is in purple. F) Example nearest neighbor microenvironment from GSK3β. Fragment 241 of the bound PDB ligand 679 is in purple. The percent sequence identity between GSK3β and aPKC is in parentheses. Proteins are shown in cartoon representation with microenvironments as semi-transparent spheres. Microenvironment color scheme is arbitrary but consistent between panels. Side chains corresponding to microenvironments are shown in stick representation. Ligands are also drawn in stick representation.

The various isoforms of atypical protein kinase C are involved in cancer development and progression [41], [42]. While elevated aPKC levels correlate with chemotherapy resistance [43], depleted or inactivated aPKC can improve chemotherapy response [44], making aPKC an attractive drug target. When applied to the kinase domain of aPKC bound to adenine (PDB ID: 4DC2 [45]), FragFEATURE predicted multiple fragments that deviated from the protein's natural ligand, ATP. Most significantly, five microenvironments proximal to the adenine moiety predicted fragment 1049 with a p-value of 9.9×10^−15^ (Figure 6A). The contributing microenvironments originated from residues Ala271, Tyr324, and Val325. We identified an alternate structure of aPKC bound to PDB ligand C58 (PDB ID: 3ZH8 [46]), a novel small molecule inhibitor of aPKC (IC50: 86 nM). Fragment 1049 is a substructure of C58 and is in close proximity to the corresponding microenvironments from 3ZH8 (Figure 6B).

In a spatially distinct part of the pocket, a set of microenvironments arising from residues Arg273, Thr385, and Asp386 predicted fragment 241 with a p-value of 9.1×10^−4^ (Figure 6D). We structurally validated this fragment using a crystal structure of aPKC bound to BI1 (PDB ID: 1ZRZ [47]). BI1 is a weak aPKC inhibitor (IC50: 3.5 µM) that contains fragment 241 as a substructure. In the crystal structure of aPKC bound to BI1, fragment 241 is in proximity to the microenvironments predicted to bind it (Figure 6E). The two fragment predictions for aPKC thus correspond to two unique inhibitor compounds. These fragments demonstrate the ability of FragFEATURE to make multiple independent fragment predictions that can serve as starting points in drug discovery for a protein target. We observe similar independent fragment predictions for protein kinase A (Text S6) (Figure S13 and S14).

To generate these fragment predictions, FragFEATURE primarily uses knowledge base information from other serine/threonine kinases as well as tyrosine kinases (Table S10). On average, aPKC shares 22% (jFATCAT) or 29% (DaliLite) sequence identity with these structures (Table S11). Unsurprisingly, multiple nearest neighbor microenvironments arise from cAMP-dependent protein kinase (PKA). PKA and aPKC are both members of the AGC Ser/Thr protein kinase family. Surprisingly, multiple nearest neighbor microenvironments also arise from glycogen synthase kinase 3 beta isoform (GSK3β) from the CMGC Ser/Thr protein kinase family. Microenvironments from Val325 and Asp386 of aPKC when compared to knowledge base members of the same microenvironment type (∼60,000) retrieve microenvironments from GSK3β as one of the five nearest non-homologous neighbors (Figure 6C and 6F). These microenvironments are located at opposite ends of the protein pocket, suggesting extended pocket similarity between aPKC and GSK3β. Interestingly, protein kinase C inhibitors bisindolylmaleimide I (BI1) and the structurally related bisindolylmaleimide IX (Ro 318220) have a side effect of stimulating glycogen synthesis [48]. After significant research, inhibition of GSK3β was identified as the mechanism of action [49], [50]. FragFEATURE's unexpected double retrieval of GSK3β as a nearest neighbor correlates well with experimental findings. This example demonstrates the identity of nearest neighbor proteins found by FragFEATURE can have interesting implications for the specificity of fragments/drugs for a pocket.

## Discussion

In this study, we demonstrate a novel approach for *in silico* fragment prediction given a protein structure of interest. FragFEATURE uses a knowledge base of local structural environments linked to ligand fragments. There are two classes of ligand fragments: “full substructure set” contains all possible substructures, “molecule parts set” contains non-overlapping substructures. Existing data-driven fragment binding predictors use the latter because it generates a tractable number of chemically intuitive parts that are assumed to be the chemically important fragments. Intuitive parts could result from dividing a molecule into ring systems, substituents, and linkers [24]. However, doing so on compounds such as those used in the exotoxin A example to predict benzamide would not yield the benzamide fragment in all cases, obscuring the underlying protein-benzamide interaction pattern. We circumvent this issue by using ligand fragments resembling a “full substructure set.” Similar to Lipinski's “rule of five” [51] that describes small molecule properties correlated with oral bioavailability, there is a “rule of three” for fragments [52]; fragment hits identified through fragment screening tend to satisfy molecular weight <300 Daltons, the number of hydrogen bond donors is ≤3, the number of hydrogen bond acceptors is ≤3, and ClogP is ≤3. However, many successful fragments violate the “rule of three” [53]. Additionally, fragments in the “rule of three” refer to compounds at least 120 Daltons in molecule weight, which is larger than the fragments in FragFEATURE's knowledge base. Thus, we do not employ any filters and include in the knowledge base all fragments that are a substructure of a PDB ligand.

However, the relative frequency of these fragments varies significantly in the knowledge base. Rare fragments do not pose a problem to FragFEATURE because it uses structural similarity to make predictions. This enables prediction of rare fragments if the query microenvironments are similar to knowledge base microenvironments binding the rare fragments. Indeed, the predicted inhibitor fragments in the presented case studies are all relatively rare. FragFEATURE filters out extremely rare fragments, such as fragments occurring a single time in the knowledge base. We observed inclusion of such data-poor fragments increased the number of incorrect predictions (Text S1) (Figure S5A). FragFEATURE therefore excludes extremely rare fragments, even though their inclusion can increase the number and diversity of fragment hypotheses.

FragFEATURE's strength lies in predicting protein-fragment associations previously observed in the PDB, though there are multiple ways to derive new associations (Text S7). Rare microenvironments are those infrequently observed in ligand-binding pockets. They pose a problem because they are likely to be dissimilar to most knowledge base microenvironments. Lack of similarity between query and knowledge base microenvironments is more likely to produce incorrect predictions. We observed low precision with the thiamin-binding proteins because the nearest neighbors of the thiamin-binding microenvironments are not very similar, compared to the nearest neighbors of the microenvironments binding the other validation ligands (Figure S15). Of course, fragment information from more dissimilar neighbors is less reliable. In this data poor scenario, using fragment information from fewer neighbors might mitigate the impact of distant “contaminating” microenvironments. We have found that the microenvironment similarity score generally enriches for good neighbors rather than achieving perfect order (Text S1) (Figure S5A). Rare microenvironments are thus a weakness of FragFEATURE; with time and more protein-ligand complexes, the performance will improve. Moreover, FragFEATURE only predicts fragments bound by the nearest neighbors. Thus, if all nearest neighbors bind natural ligands, FragFEATURE (as currently configured) will not predict a drug-related fragment even if drugs are known to bind at that protein site. Future work may focus on enriching FragFEATURE predictions for drug-like fragments.

Using the PDB-derived microenvironment-fragment associations, FragFEATURE showed strong ability to predict fragments corresponding to known ligands of a structure. These statistically significant fragment predictions imply repeated observation of microenvironment-fragment interaction patterns but do not imply high affinity binding. This is a limitation stemming from how FragFEATURE extracts fragment information from PDB ligands (Text S8). Nevertheless, FragFEATURE achieved very high recall for nucleotide and non-nucleotide ligands as a general, non-ligand-specific method. Notably, it uses fragment information from nearest neighbor proteins with low sequence identity to the test structure. In rare cases, high sequence identity is observed (Figure S9) due to FragFEATURE's usage of the PDB's pre-computed 50% sequence identity clusters. The PDB's sequence identity clusters do not group proteins with significantly different lengths. On occasion, such proteins can possess high sequence identity over the length of the shorter protein or over a local region (i.e. shared domain), leading to the observation of high sequence identity between a test structure and nearest neighbor protein. FragFEATURE also predicted fragments for ligands bound by the structure (holo predictions) and ligands/inhibitors known to bind the structure (apo predictions). Apo fragment predictions are especially important as they reflect the most likely use case. As performance decreased more for the ligand-free structures than ligand-bound structures, pocket conformation is a key consideration. In other work, we have demonstrated improvement in protein functional site recognition using protein structure ensembles from molecular dynamics [54], [55]. Likewise, fragment prediction on conformational ensembles should further improve FragFEATURE's predictive capabilities.

FragFEATURE performance also depends on the definition of the ligand-binding pocket. As we use pocket microenvironments to search the knowledge base for preferred fragments, both missing and extraneous microenvironments can lead to false negative and false positive predictions. We used fPocket, a good algorithm for large-scale pocket prediction, but it struggled to identify the ligand-binding site in ligand-free structures. Ligand-free protein conformations make pocket detection difficult and incorporating structural ensembles from molecular dynamics may improve pocket detection. fPocket defines relatively large pockets, producing fragment predictions in protein regions with no bound ligand in the analyzed structure or homolog structures. These predictions are very hard to evaluate because we lack evidence of binding to these extended regions. Use of pocket refinement algorithms like SURFNET-ConSurf [56] could improve pocket definition and thereby focus and improve fragment binding prediction. It uses evolutionary conservation information from the ConSurf-DB [57] to trim predicted pockets to regions proximal to conserved residues. Given that pockets with conserved residues likely have conserved structure, such pockets might be easier tests cases for FragFEATURE. Hence, to avoid biasing performance, we did not perform pocket trimming.

Two additional considerations for FragFEATURE performance are microenvironment independence and multiple hypothesis testing. FragFEATURE assumes microenvironment independence when calculating Fisher's p-values for fragments of a microenvironment set. We determined this to be reasonable even for spatially proximal microenvironments (Text S9). Additionally, there is testing of multiple fragments and microenvironment sets for statistically significant fragments. Such multiple hypothesis testing normally requires p-value correction, but we argue it to be largely unsuitable and unnecessary in the context of this work (Text S10).

In summary, we present a novel approach to fragment binding prediction that differs from existing predictors in multiple respects. FragFEATURE uses a knowledge base of all available microenvironment-fragment interactions, retaining information contained in common and rare interactions. By using machine learning to compare a query protein pocket to the knowledge base to identify statistically preferred fragments, FragFEATURE performs pattern recognition on the fly. However, unlike many machine learning approaches, the method is transparent: predictions can be traced back to individual microenvironment-fragment associations. FragFEATURE is also hypothesis-free, requiring no initial input of fragments to test. It provides the fragments similar protein pockets bind, rather than estimates of fragment complementarity. We show FragFEATURE predicts fragments corresponding to bound ligands or ligands known to bind, where the ligands include endogenous compounds with some drug-like compounds. It is important to note that many drugs such as ATP mimetic kinase inhibitors [58] possess parts of the native ligand and thus accurate prediction of such fragments is important.

Importantly, FragFEATURE requires no prior knowledge of ligands (endogenous or synthetic) that interact with the protein target. It relies solely on the target's 3D structure and can be applied to any protein with a solved structure or high quality homology model. Additionally, the structural space of protein pockets is small, with the PDB showing good coverage of pocket space [59]. DrugFEATURE, a related method that quantifies a protein pocket's druggability, has also shown druggable pockets to be composed of microenvironments from known drug-binding sites in the PDB [60]. Collectively, FragFEATURE's reliance on structural information, good coverage of pocket space by the PDB, and shared microenvironments between druggable sites and known druggable sites position FragFEATURE to possess an advantage in studying less characterized proteins.

Other methods for identifying compounds for a protein target include using information from (1) known bioactive compounds for the target, (2) compounds that bind to homologs of the target, and (3) compounds that bind to proteins non-homologous to the target but share binding specificity (i.e. bind similar endogenous ligands as the target). Such compounds can serve as valuable starting points in drug discovery [61], but there is also a weakness associated with each approach. First, a protein target's binding site can be promiscuous and have the ability to bind dissimilar ligands [62]. Focusing on fragments from known bioactive compounds may limit the scope of discovery. Second, numerous proteins show global structural similarity but dissimilar ligand-binding pockets [59]. Proteins homologous to a target may therefore have different pockets and thereby bind different ligands, a behavior already observed with protein kinases and their inhibitors [50]. Lastly, a single ligand can be associated with multiple types of binding environments (binding modes) [63]. Bioactive compounds taken from proteins binding the same endogenous ligand may therefore have limited relevancy. As a knowledge-based method, FragFEATURE inherently captures these complex relationships between proteins and ligands to the extent that they are represented in the PDB. It identifies the relevant protein-ligand relationships on a structure-by-structure basis. It can thus be a complementary tool in understanding the relevancy of ligand fragments taken from prior knowledge. Fragments found by both FragFEATURE and existing strategies may deserve higher priority. As FragFEATURE returns fragment predictions with probability estimates, it is also possible to incorporate user-defined prior probabilities for the fragments to be used in calculating refined posterior estimates.

Fragments predicted by FragFEATURE may overlap with available binding information for the target or be intuitive to a researcher with expertise in structure-based or fragment-based drug design. However, FragFEATURE provides a systematic statistical framework within which to interpret a fragment prediction along with the structural evidence for the prediction. FragFEATURE uses fragment information from proteins with low sequence identity to the target protein. The retrieved nearest neighbor protein structures may highlight unexpected local structural similarity. Shared fragment preferences between proteins can provide an opportunity for polypharmacology [64] or highlight potential off-target complications [65]. The fragments bound by these nearest neighbors may also suggest variations of a fragment that could alter potency or specificity. FragFEATURE as a knowledge-based method provides a means to understand the binding mode of a fragment across unique proteins. As a systematic and quantitative approach, it complements and supplements existing approaches and human intuition in the drug discovery pipeline.

## Methods

### Feature microenvironments

The FEATURE software [27] captures the physicochemical information around a point of interest by segmenting the local environment into six concentric shells, each of 1.25 Å in thickness (Figure S1A). Within each shell, FEATURE evaluates 80 physicochemical properties including atom type, residue class, hydrophobicity, and secondary structure. It ignores all heteroatom information. This enables conversion of a local structural environment into a numeric vector of length 480 (6 shells×80 properties) (Figure S1B). These local structural environments are termed microenvironments.

Microenvironment centers correspond to residue side chains or backbone atoms. For the side chain centers, we use those defined by PocketFEATURE [66], a FEATURE-based pocket similarity algorithm. A center is either a physical atom location (e.g. alanine beta carbon, ALA.CB) or the midpoint of multiple physical atom locations (e.g. center of phenylalanine benzene ring, PHE.PSEU). We use PSEU to indicate a pseudo atom location. There are twenty-one microenvironments originating from amino acid side chains (from PocketFEATURE), twenty microenvironments originating from the backbone oxygen, and nineteen microenvironments originating from the backbone nitrogen (Figure S1C). We group microenvironments from the backbone into a general oxygen type (RES.O) and nitrogen type (RES.N). There are thus 23 microenvironment types.

### Microenvironment similarity

To determine microenvironment similarity, we adopted the approach used by PocketFEATURE [66]. PocketFEATURE first derives the background variation of microenvironment properties and uses this to calculate a Tanimoto similarity coefficient between a pair of FEATURE vectors. Similar to the protocol used in PocketFEATURE, we define background variation as the observed variation in microenvironment properties across non-redundant proteins. We measure it by calculating the standard deviation of each property in a microenvironment type's non-redundant set. The non-redundant set of a microenvironment type contains microenvironments selected from proteins with less than 95% sequence identity. In making the non-redundant set for the backbone oxygen type (or nitrogen type), we include 500 non-redundant microenvironments from each of the 20 (or 19) contributing residues.

We use PocketFEATURE's Tanimoto coefficient to perform similarity comparisons between microenvironments of the same type (e.g. backbone oxygen to backbone oxygen). This Tanimoto coefficient measures the similarity between a pair of microenvironment vectors (**x** and **y**) as the ratio of shared properties to non-zero properties. Like PocketFEATURE, we define shared properties as those whose difference is less than the standard deviation of the property. If *c* is the number of shared properties and *a* and *b* the number of non-zero properties in vector **x** and **y** respectively, the Tanimoto coefficient (Tc) is as follows: 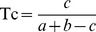


### Ligand fragmentation

Each PDB ligand is fragmented into overlapping substructures ranging from three to thirteen heavy atoms in size, which we regard as fragments of the ligand (Figure S3). We first retrieve the ligand substructures using the PubChem superstructure search function in PUG SOAP, a web services access layer to PubChem functionality. Default parameters are used in addition to hydrogen stripping of the ligand and prohibiting single and double bonds to match aromatics. We filter the retrieved substructures (fragments) for size (three to thirteen heavy atoms) and compute the atom-to-atom mapping between the fragments and the ligand using SMSD [67]. This enables the creation of a fragment list for each ligand heavy atom.

### Knowledge base construction

From the PDB (01/01/2013 snapshot), we retrieve all protein X-ray crystallography structures with resolution less than 3 Å. We then select the structures bound to small molecules, ignoring small molecules less than three heavy atoms, greater than 100 heavy atoms, or corresponding to commonly used buffers and crystallization agents. PDB ligands with over 100 heavy atoms tended to produce errors with either PUG SOAP or SMSD. For the resulting protein-ligand complexes, we calculate the location of all pseudo atoms (see Feature Microenvironments). We then use a distance cutoff to identify protein atoms/pseudo atoms “interacting” with the ligands, keeping side chain atoms/pseudo atoms within 5 Å and backbone atoms within 4 Å of a ligand heavy atom. The shorter distance cutoff for the backbone atoms helps exclude atoms participating in intra-protein hydrogen bonding and thus not involved in ligand binding. We converted the collected protein atoms/pseudo atoms into microenvironments using FEATURE and divided them into 23 types (see Feature Microenvironments). By definition, each microenvironment is “interacting” with one or more ligand heavy atoms. For each ligand heavy atom interacting with a microenvironment, we retrieve its associated fragment list and take the union of these lists as the microenvironment's associated fragments (Figure S3). This produces the link between microenvironments and fragments (Figure 1).

### Fragment prediction for single microenvironments

Given a query microenvironment, we compare it to knowledge base microenvironments of the same type. This allows us to sort the knowledge base by similarity to the query using the Tanimoto coefficients. We then filter the knowledge base for homology by first removing microenvironments from proteins homologous to the query. Of the remaining microenvironments, we keep only the most similar microenvironment for microenvironments from homologous proteins. This ensures the final knowledge base microenvironments are non-homologous to the query and to each other. We define homology as ≥50% sequence identity as pre-computed by the PDB. This produces a query-specific non-homologous knowledge base ordered by microenvironment similarity to the query.

To determine the fragment binding preferences of the query microenvironment, we take the *k* most similar non-homologous microenvironments (*k* nearest neighbors) and their corresponding fragment list. For each fragment in these lists, we calculate a hypergeometric p-value. This requires the number of neighbors selected (*k*), the number of neighbors binding the fragment (*m*), the number of microenvironments in the non-homologous knowledge base binding the fragment (*M*), and the number of microenvironments in the non-homologous knowledge base (*N*). The hypergeometric p-value (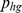) for a fragment is as follows: 
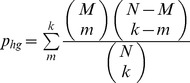


This p-value varies with different values of *k* and different fragment inclusion thresholds for *M*. We tested a grid of values where 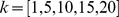 and 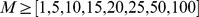 and set 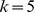 and 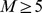 (Text S1).

### Performance of fragment prediction for single microenvironments

For each of the 23 microenvironment types in the knowledge base, we perform fragment prediction on the microenvironments of the non-redundant set (see Microenvironment Similarity). We take the most significant fragment predicted for a microenvironment and compare it to the microenvironment's fragment list to determine its validity (correct/incorrect). We then use the predicted fragments' p-values and validity labels to compute a precision recall curve for each microenvironment type. The area under the precision recall curve (AUPR) serves as a measure of overall performance. An AUPR approaching 1.0 indicates similar microenvironments bind similar fragments.

### Validation data set

We selected eight ligands to validate FragFEATURE's performance: adenine (ADE), adenosine-5′-diphosphate (ADP), flavin-adenine dinucleotide (FAD), nicotinamide-adenine dinucleotide (NAD), pyridoxal-5′-phosphate (PLP), triclosan (TCL), thiamine diphosphate (TPP), and thiamin (VIB). For the ligand-bound structures, we retrieved all structures from the PDB in complex with the validation ligands. For the ligand-free structures, we retrieved all protein-only structures from the PDB homologous (95% sequence identity) to the ligand-bound structures.

### Pocket detection

Three types of pockets are of interest: observed ligand-binding pockets, predicted pockets from ligand-bound structures, and predicted pockets from ligand-free structures. An observed ligand-binding pocket contains the side-chain atoms within 5 Å and backbone atoms within 4 Å of any validation ligand heavy atom. A predicted pocket for a ligand-bound or ligand-free structure is the atoms forming the largest pocket predicted by fPocket. fPocket performs pocket detection for each protein chain individually with the ligand information removed. Protein atoms comprising the different pockets are then passed to FEATURE to calculate their microenvironments. Microenvironments centered on a pseudo atom are calculated if any of the atoms contributing to the pseudo atom location are part of the pocket (see FEATURE Microenvironments).

### Microenvironment sets within a protein pocket

Spatially proximal microenvironments may have similar fragment preferences due to their proximity. Thus, for a microenvironment in a pocket, we retrieve all microenvironments within 5.5 Å to create a set of *N* spatially proximal microenvironments. However, given that some microenvironments in the set may not be informative for fragment prediction, we also create all subsets of size *N*–1 to 2 microenvironments (Figure S6). Repeating this procedure for each microenvironment in the pocket produces all combinations of spatially proximal microenvironments. We use these combinations to search for enriched fragment preferences across the included microenvironments.

### Fragment prediction for microenvironment sets

Each microenvironment of a microenvironment set has fragment preferences represented as a list of fragment hypergeometric p-values (see Fragment Prediction for Single Microenvironments). Fragments with a hypergeometric p-value for all microenvironments within a set are consensus fragments regardless of the hypergeometric p-value. We keep only consensus fragments to ensure agreement and coherence among the microenvironments of a set. For each consensus fragment, we use Fisher's method to combine the hypergeometric p-values (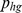) from *n* microenvironments, calculating the following test statistic that follows the chi-squared distribution with 2*n* degrees of freedom: 
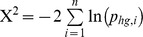


This aggregates a fragment's hypergeometric p-values into a single Fisher's p-value, resulting in a Fisher's p-value ranked list of fragments for each microenvironment set (Figure 2). We remove non-significant fragments using an acceptance threshold of 10^−2^. We also remove microenvironment sets contained within a larger set that has a better p-value because they do not contain new information.

### FragFEATURE recall and precision

Recall measures the fraction of the bound ligands predicted by the fragments. We treat each validation ligand as a set of chemical moieties (Figure S7). Bound moieties are those with at least three pocket microenvironments in proximity (within 5 Å of side chain microenvironments or within 4 Å of backbone microenvironments). For the ligand-free structures, we infer bound moieties using a homologous (95% identical) ligand-bound structure. A moiety is predicted if a predicted fragment contains more than 50% of the moiety's atoms. Recall is thus the fraction of bound moieties recaptured by the predicted fragments: Recall = # predicted moieties/# bound moieties

Precision is the ratio of correct fragment predictions to total fragment predictions. We identify correct fragments using ligand information from both the structure analyzed (query) and the structures homologous to the query. This helps take into account that proteins may bind multiple ligands at a given site. We retrieve PDB structures sharing at least 95% sequence identity to the query and use BLAST+ [68] to align their protein sequences to the query. This provides a residue-to-residue mapping between the query and each homolog such that the query microenvironments can be mapped to corresponding atom or pseudo atom locations in the homolog structures.

Fragment predictions arise from microenvironment sets and are correct, incorrect or no information (Figure S8). Correct fragments are in proximity to one or more of the microenvironments in the microenvironment set, in either the query structure or a homolog structure. Proximity refers to a maximal distance of 5 Å to a side chain microenvironment or 4 Å to a backbone microenvironment. Incorrect fragment predictions conflict with the fragments in proximity to the microenvironments of the set. “No information” fragment predictions are those in regions of a protein where no structure has ligand-binding information. The validity of these predictions is unclear and thus we exclude them. Precision is thus: Precision = # correct fragment predictions/# fragment predictions

### Sequence identity of nearest neighbors

Each fragment prediction is made by a set of microenvironments with each microenvironment using fragment information from the five nearest non-homologous neighbors. A subset of these nearest neighbors contributes to the fragment prediction (e.g. bind the predicted fragment). We calculate the percent sequence identity between the query structure and this subset of nearest neighbor protein structures. We analyze the most significant correct fragment prediction for each test structure, so test proteins without a correct fragment prediction are excluded from analysis. To determine the percent sequence identity between a pair of proteins, we use BLAST+ to align their amino acid sequences. We consider alignments that fail to cover at least 20% of the smaller protein or that possess an alignment E-value ≥10 (default) as failed alignments.

### FragFEATURE computation time

Computation time varies with the size of the pocket of interest as measured by the number of microenvironments comprising the pocket. The process begins with an input list of atoms defining the pocket and ends with an output file of fragment predictions. The ligand-binding pockets generally possess five to fifty-five microenvironments. We select eleven pocket sizes to cover this range evenly and time five pockets for each pocket size to obtain computation time as a function of pocket size (Figure S16). Computations are performed on an Intel Xeon 2.6 GHz based system.

## Supporting Information

Figure S1 FEATURE microenvironments.
(DOCX)

Figure S2 Breakdown of knowledge base microenvironments.
(DOCX)

Figure S3 Ligand fragmentation.
(DOCX)

Figure S4 Breakdown of knowledge base fragments.
(DOCX)

Figure S5 Fragment prediction for single microenvironments.
(DOCX)

Figure S6 Microenvironment set definition.
(DOCX)

Figure S7 Chemical moieties of the validation ligands.
(DOCX)

Figure S8 Fragment evaluation.
(DOCX)

Figure S9 Sequence identity between test proteins and nearest neighbor proteins.
(DOCX)

Figure S10 Comparison of observed ligand binding pockets and predicted pockets.
(DOCX)

Figure S11 FragFEATURE performance on predicted pockets.
(DOCX)

Figure S12 Fragment prediction and validation for Abl tyrosine kinase.
(DOCX)

Figure S13 Fragment prediction and validation for protein kinase A (PKA).
(DOCX)

Figure S14 Alternative fragments for PDB ligands I5S, M77, and TZ1.
(DOCX)

Figure S15 Microenvironment similarity of nearest neighbors.
(DOCX)

Figure S16 FragFEATURE computation time.
(DOCX)

Table S1 Prevalence of validation ligands across 50% sequence identity clusters.
(DOCX)

Table S2 Breakdown of protein pockets tested.
(DOCX)

Table S3 PDB ligands supporting the benzamide prediction for exotoxin A.
(DOCX)

Table S4 PDB structures supporting the benzamide prediction for exotoxin A.
(DOCX)

Table S5 Protein names of PDB structures supporting the benzamide prediction for exotoxin A.
(DOCX)

Table S6 Sequence identity between PDB structures supporting the benzamide prediction for exotoxin A.
(DOCX)

Table S7 PDB structures supporting fragment 13509097/benzamide prediction for DAPK1.
(DOCX)

Table S8 Sequence identity of PDB structures supporting fragment 13509097/benzamide prediction for DAPK1.
(DOCX)

Table S9 PDB ligands supporting the benzamide prediction for DAPK1.
(DOCX)

Table S10 PDB structures supporting fragment 1049/241 prediction for aPKC.
(DOCX)

Table S11 Sequence identity of PDB structures supporting fragment 1049/241 prediction for aPKC.
(DOCX)

Text S1 Similar microenvironments bind similar fragments.
(DOCX)

Text S2 FragFEATURE predicts fragments of bound ligands using predicted protein pockets.
(DOCX)

Text S3 FragFEATURE predicts fragments of unbound ligands using predicted protein pockets.
(DOCX)

Text S4 Inhibitor fragment predictions for cholix toxin.
(DOCX)

Text S5 Inhibitor fragment predictions for Abl tyrosine kinase.
(DOCX)

Text S6 Inhibitor fragment predictions for protein kinase A.
(DOCX)

Text S7 Expanding the knowledge base beyond the information in the PDB.
(DOCX)

Text S8 FragFEATURE and ligand/fragment affinity.
(DOCX)

Text S9 Microenvironment independence assumption.
(DOCX)

Text S10 Multiple hypothesis correction.
(DOCX)
