# Supplementary material for: Knowledge-based Fragment Binding Prediction
Source: PLoS Comput Biol. 2014 Apr 24;10(4):e1003589. doi: 10.1371/journal.pcbi.1003589 (PMC3998881; doi:10.1371/journal.pcbi.1003589)
Supplement: Figure S1 — FEATURE microenvironments. (DOCX) [file pcbi.1003589.s001.docx]

**Figure S1. FEATURE microenvironments**


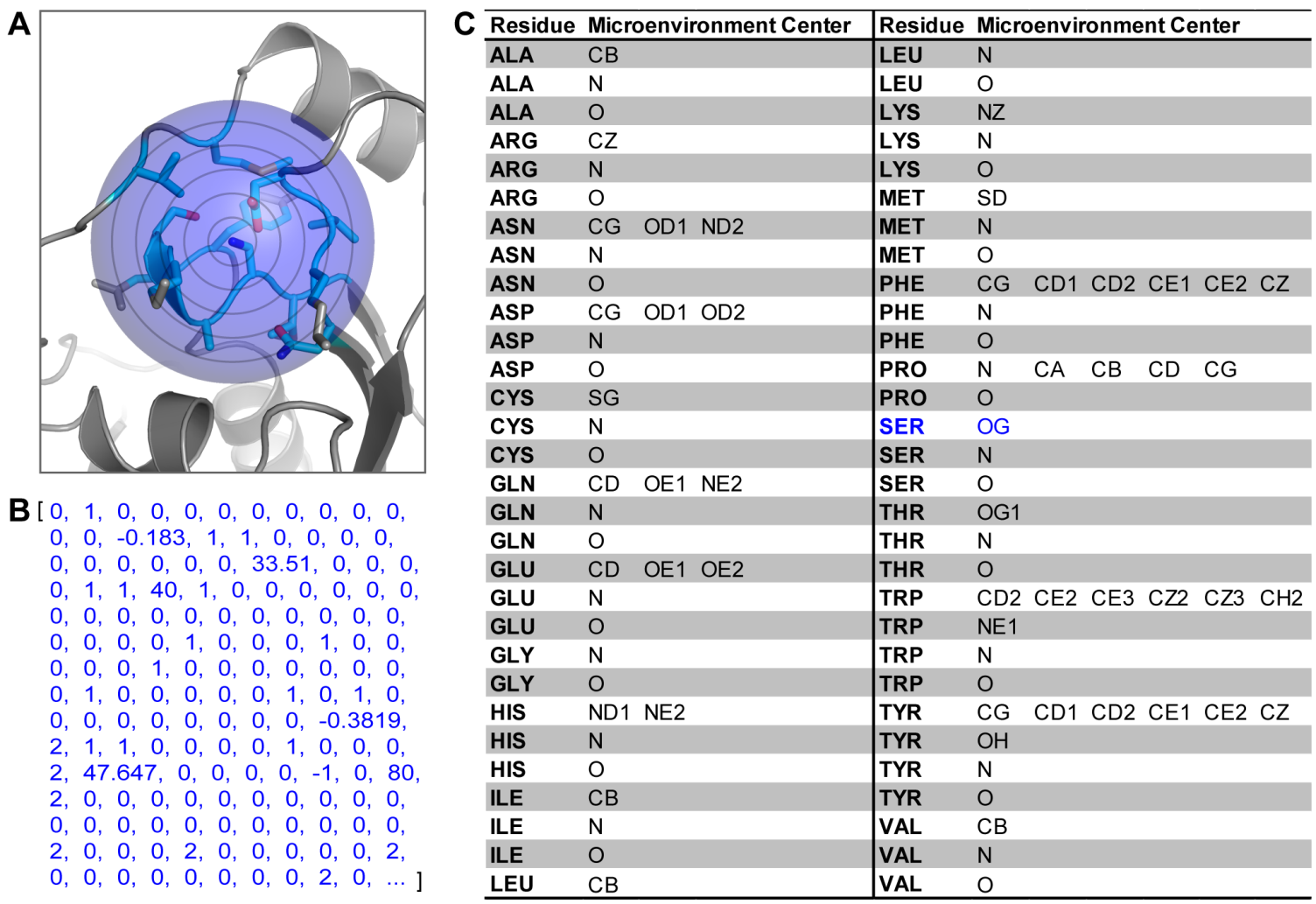


1. FEATURE microenvironment. Each microenvironment has six concentric shells centered about a point of interest, such as the serine gamma oxygen shown here.
2. FEATURE microenvironment vector.
3. FEATURE microenvironment centers. When more than one atom is listed, the midpoint of the atom positions is taken as the microenvironment center.
