# Supplementary material for: Knowledge-based Fragment Binding Prediction
Source: PLoS Comput Biol. 2014 Apr 24;10(4):e1003589. doi: 10.1371/journal.pcbi.1003589 (PMC3998881; doi:10.1371/journal.pcbi.1003589)
Supplement: Figure S2 — Breakdown of knowledge base microenvironments. (DOCX) [file pcbi.1003589.s002.docx]

**Figure S2. Breakdown of knowledge base microenvironments**


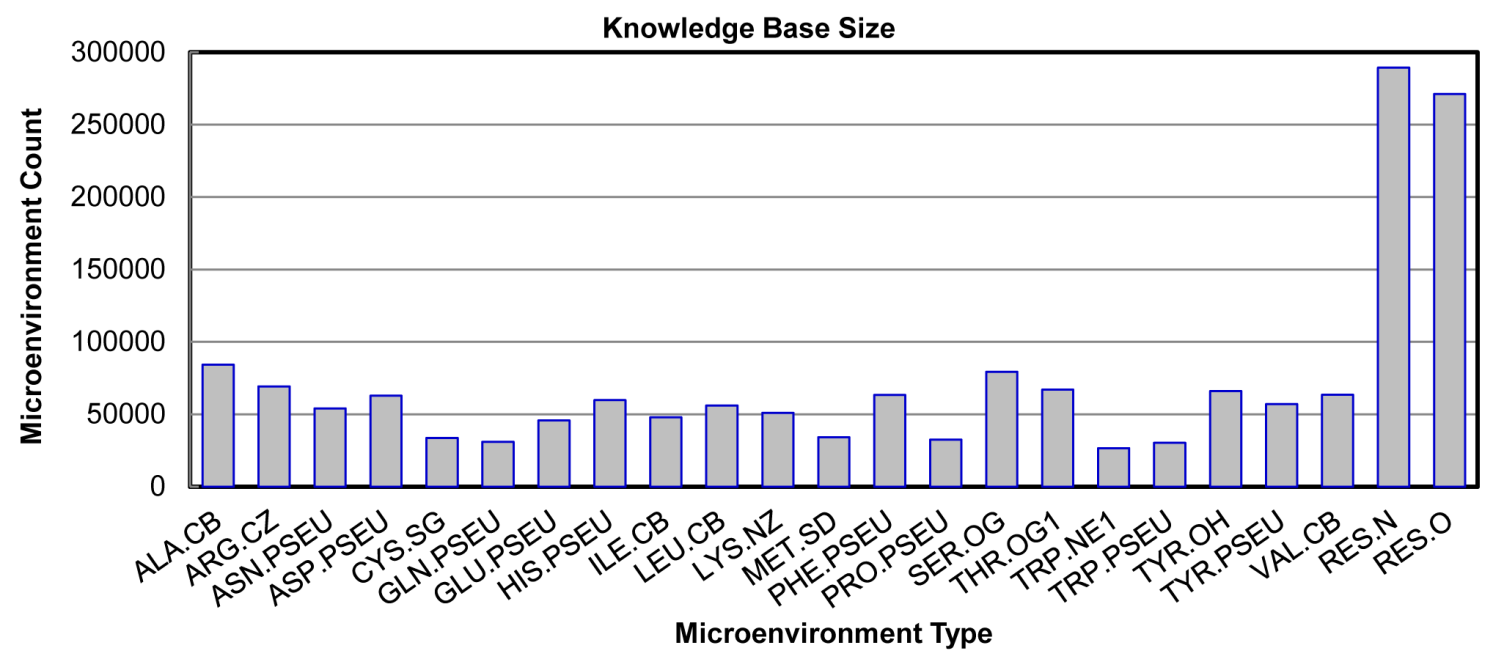


Prevalence of the microenvironment types in the knowledge base. The 23 microenvironment types in the knowledge base correspond to different functional centers, specified by their 3-letter residue code and followed by their residue atom. A 3-letter residue code of **RES** indicates a backbone microenvironment type that includes multiple residues. A residue atom of **PSEU** refers to a pseudo atom that corresponds to the average position of multiple residue atoms.
