# Supplementary material for: Knowledge-based Fragment Binding Prediction
Source: PLoS Comput Biol. 2014 Apr 24;10(4):e1003589. doi: 10.1371/journal.pcbi.1003589 (PMC3998881; doi:10.1371/journal.pcbi.1003589)
Supplement: Figure S3 — Ligand fragmentation. (DOCX) [file pcbi.1003589.s003.docx]

**Figure S3. Ligand fragmentation**

**
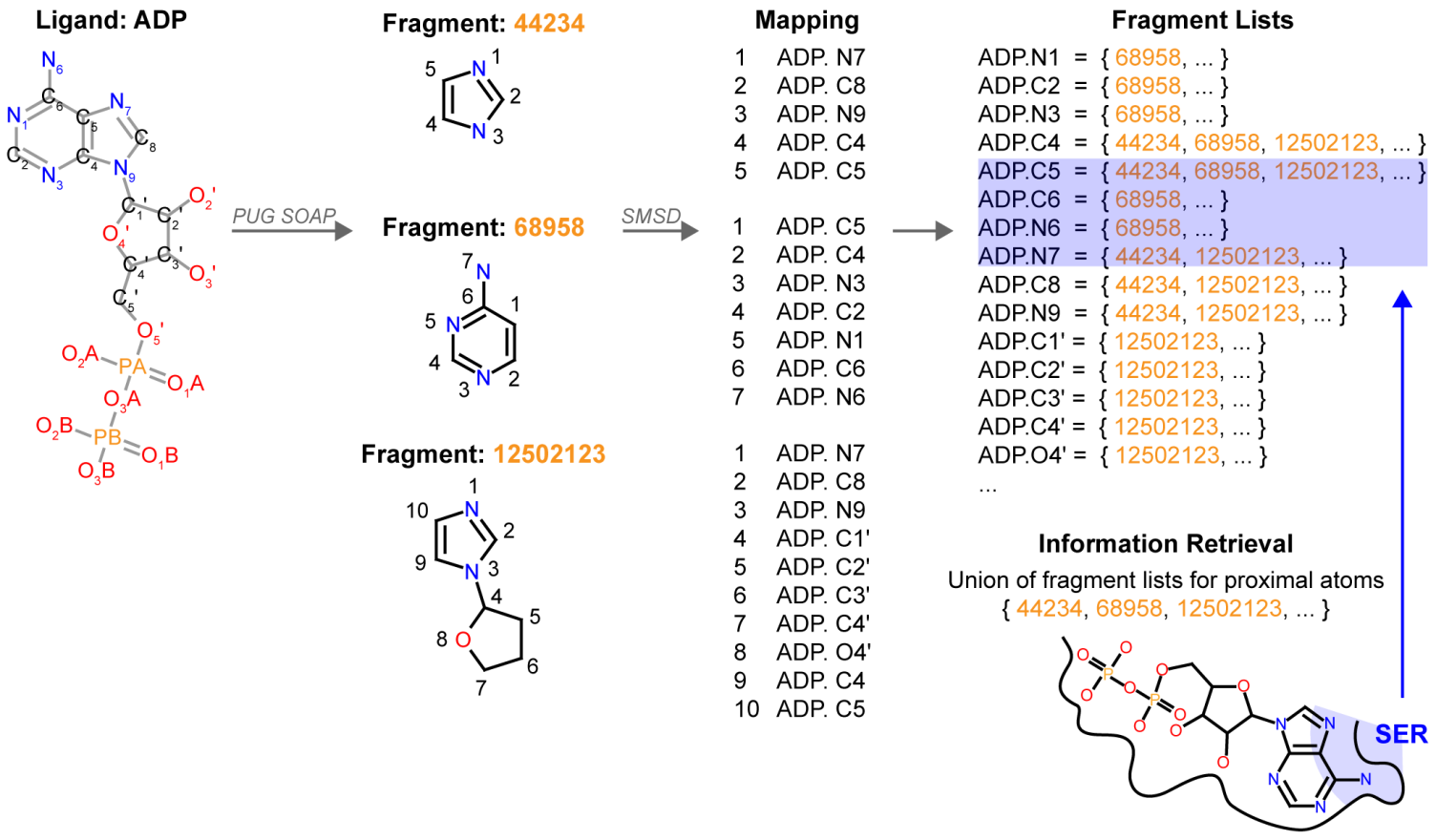
**

Diagramed is an example fragmentation of PDB ligand ADP. We pass the ADP SMILES string to PUG SOAP, which returns a list of PubChem compounds that are substructures of the query ligand. These compounds (fragments) are passed to SMSD to generate the atom-to-atom mapping between ADP and the fragments. We then post process the mapping information to produce a fragment list for each heavy atom of ADP. To determine the relevant fragments for a microenvironment, we take the union of the fragment lists for the proximal atoms.
