# Supplementary material for: Knowledge-based Fragment Binding Prediction
Source: PLoS Comput Biol. 2014 Apr 24;10(4):e1003589. doi: 10.1371/journal.pcbi.1003589 (PMC3998881; doi:10.1371/journal.pcbi.1003589)
Supplement: Figure S4 — Breakdown of knowledge base fragments. (DOCX) [file pcbi.1003589.s004.docx]

**Figure S4. Breakdown of knowledge base fragments**


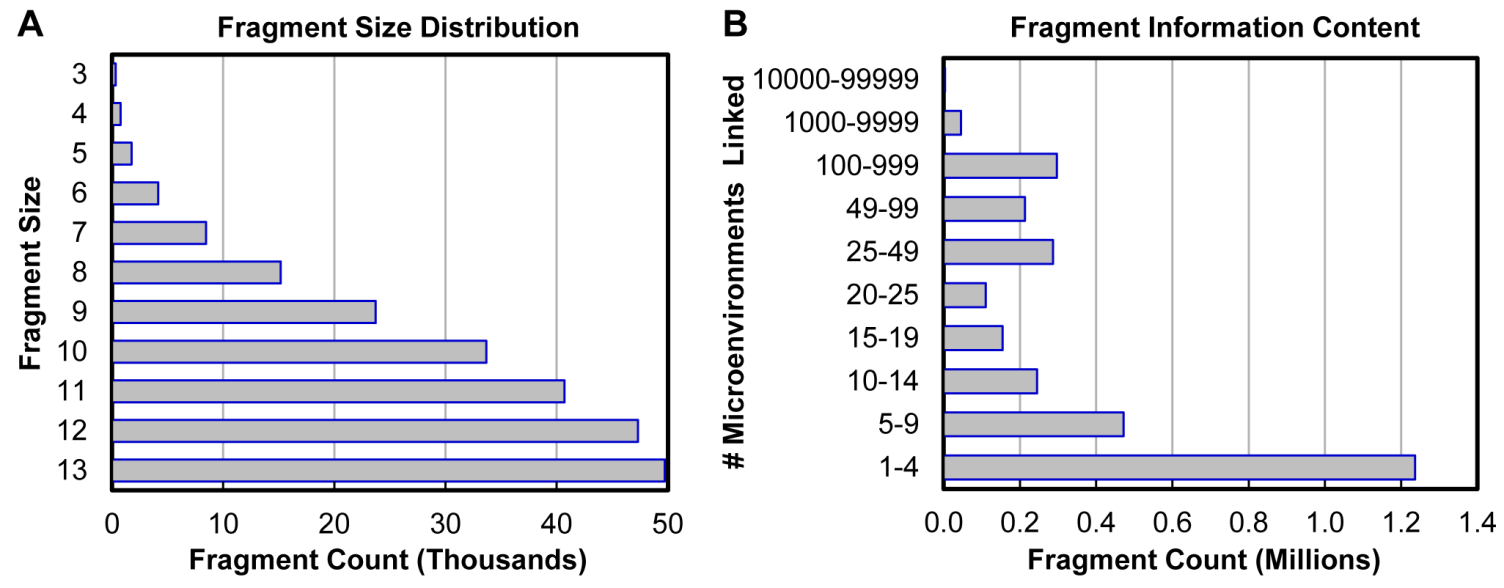


1. Fragment size distribution. The plot shows the unique set of knowledge base fragments binned by size (number of heavy atoms).
2. Fragment information content. The plot shows knowledge base fragments binned by the number of microenvironments linked to the fragment.
