# Supplementary material for: Knowledge-based Fragment Binding Prediction
Source: PLoS Comput Biol. 2014 Apr 24;10(4):e1003589. doi: 10.1371/journal.pcbi.1003589 (PMC3998881; doi:10.1371/journal.pcbi.1003589)
Supplement: Figure S5 — Fragment prediction for single microenvironments. (DOCX) [file pcbi.1003589.s005.docx]

**Figure S5. Fragment prediction for single microenvironments**


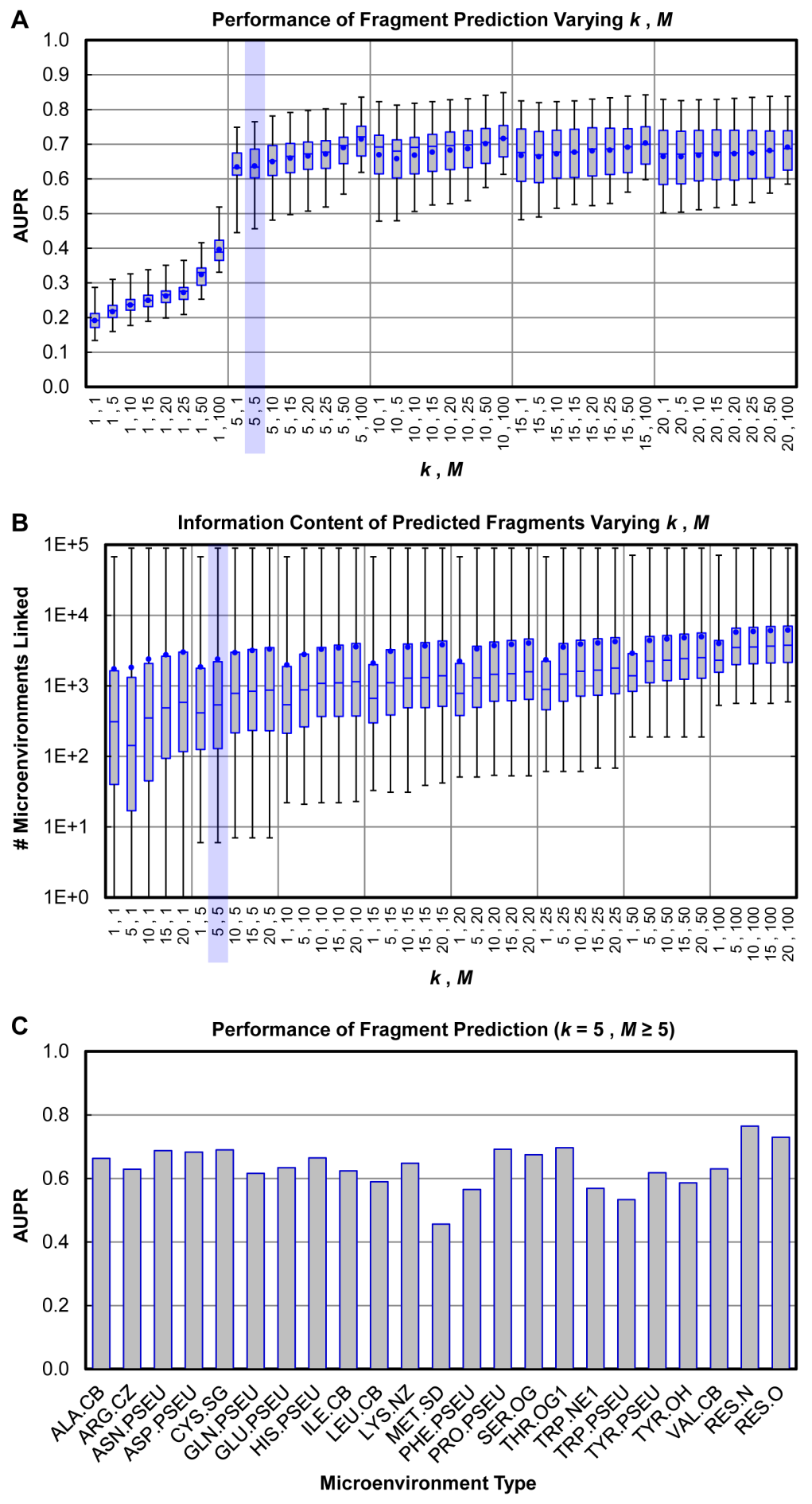


1. Shown is the performance of fragment prediction with single microenvironments for various *k* and *M*. The performance metric is the area under the precision recall curve (AUPR) across the 23 microenvironment types. An AUPR approaching 1.0 reflects an increasing number of correct fragment predictions. The boxplots show the min, first quartile, second quartile, third quartile, and max AUPR associated with a parameter pair (*k*,*M*). Blue dots represent the average AUPR. Parameter pairs are grouped by *k* with the highlighted pair representing the final selected parameters (see panel C).
2. Shown is the information content of predicted fragments for various *k* and *M*. Information content is the number of microenvironments linked to the predicted fragments across the 23 microenvironment types. The boxplots show the min, first quartile, second quartile, third quartile, and max information content of predicted fragments associated with a parameter pair (*k*,*M*). Blue dots represent the average information content of predicted fragments. Parameter pairs are grouped by *M* with the highlighted pair representing the final selected parameters (see panel C).
3. Shown is the performance of fragment prediction with single microenvironments for the 23 microenvironment types (*k* = 5 and *M* ≥ 5). The microenvironment types correspond to different functional centers specified by their 3-letter residue code and followed by their residue atom. A 3-letter residue code of **RES** indicates a backbone microenvironment type that includes multiple residues. A residue atom of **PSEU** refers to a pseudo atom that corresponds to the average position of multiple residue atoms. AUPR refers to the area under the precision recall curve. An AUPR approaching 1.0 reflects an increasing number of correct fragment predictions.
