# Supplementary material for: Knowledge-based Fragment Binding Prediction
Source: PLoS Comput Biol. 2014 Apr 24;10(4):e1003589. doi: 10.1371/journal.pcbi.1003589 (PMC3998881; doi:10.1371/journal.pcbi.1003589)
Supplement: Figure S6 — Microenvironment set definition. (DOCX) [file pcbi.1003589.s006.docx]

**Figure S6. Microenvironment set definition**

**
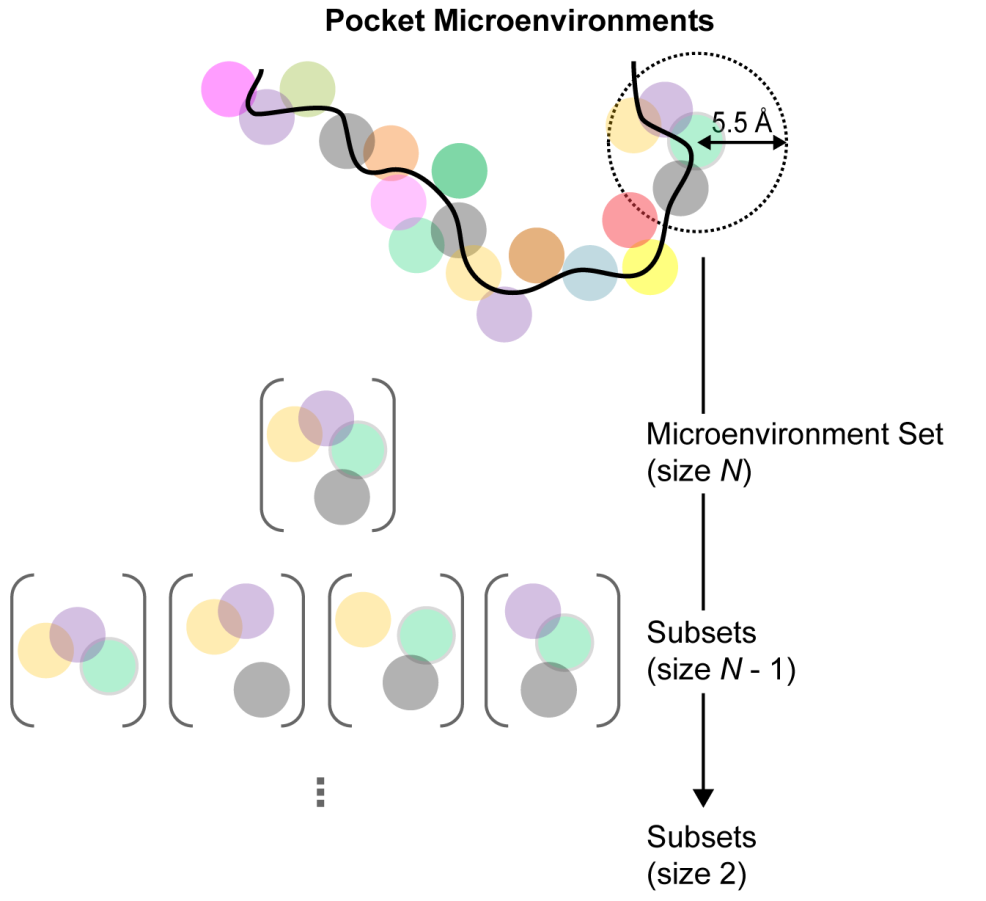
**

The centers of the pocket microenvironments are drawn as semi-transparent circles of arbitrary color. For each microenvironment, we retrieve all other microenvironments within 5.5Å. These proximal microenvironments make up a set of size *N*, from which all subsets of size *N*-1 to 2 are determined. We repeat this process for each pocket microenvironment to generate sets of proximal microenvironments for the pocket.
