# Supplementary material for: Knowledge-based Fragment Binding Prediction
Source: PLoS Comput Biol. 2014 Apr 24;10(4):e1003589. doi: 10.1371/journal.pcbi.1003589 (PMC3998881; doi:10.1371/journal.pcbi.1003589)
Supplement: Figure S7 — Chemical moieties of the validation ligands. (DOCX) [file pcbi.1003589.s007.docx]

**Figure S7. Chemical moieties of the validation ligands**


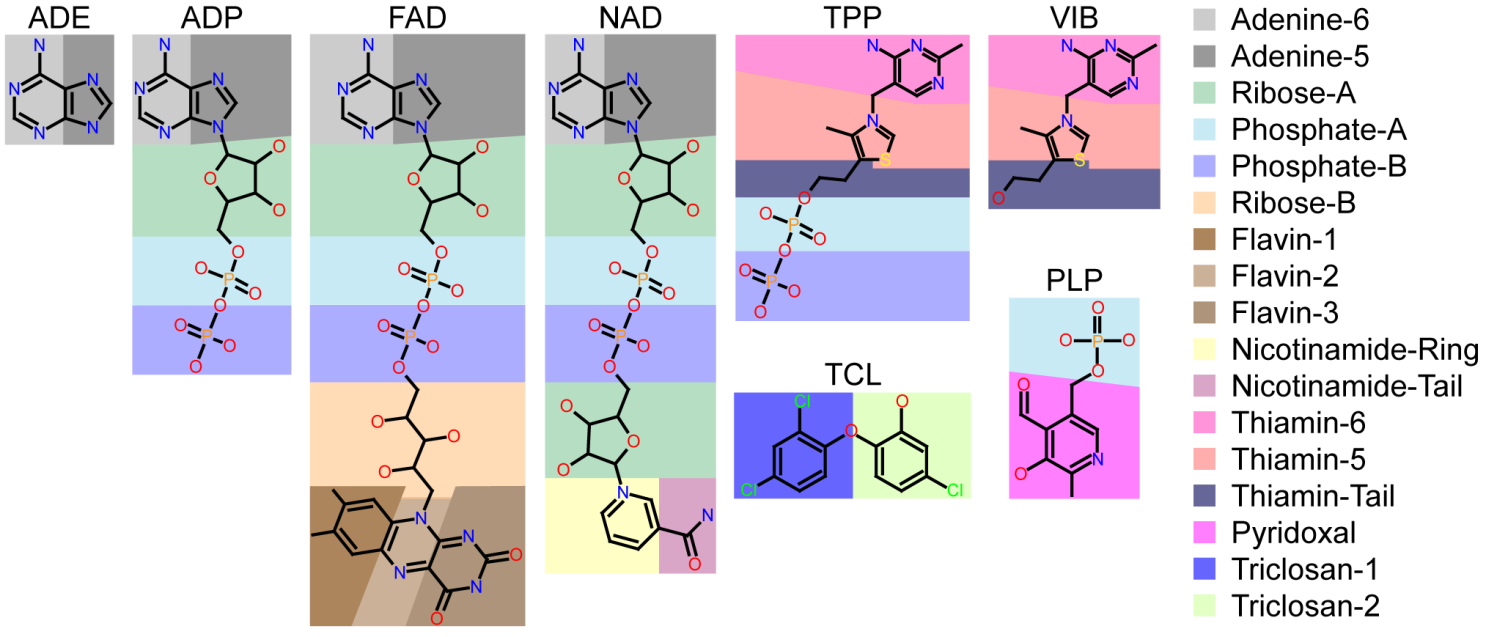


Validation ligands are divided into chemical moieties that span the length of the molecule. Moieties sharing a color are identical, though not all identical moieties share a color to maintain clarity.
