# Supplementary material for: Knowledge-based Fragment Binding Prediction
Source: PLoS Comput Biol. 2014 Apr 24;10(4):e1003589. doi: 10.1371/journal.pcbi.1003589 (PMC3998881; doi:10.1371/journal.pcbi.1003589)
Supplement: Figure S8 — Fragment evaluation. (DOCX) [file pcbi.1003589.s008.docx]

**Figure S8. Fragment evaluation**


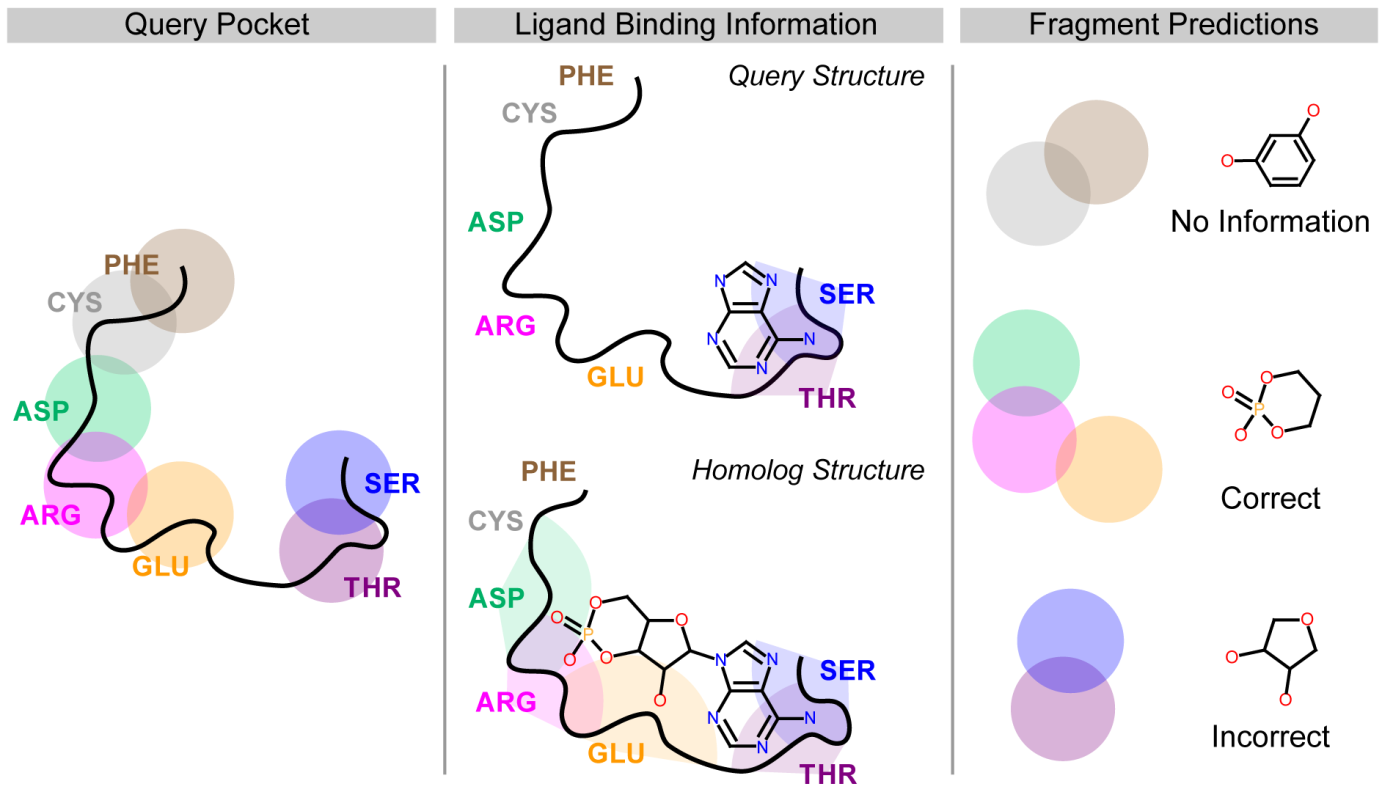


Given a query protein pocket in the form of microenvironments (semi-transparent circles) (left), FragFEATURE generates fragment predictions for different microenvironment sets within the pocket (right). Using fragment information from both the query structure and ligand-bound homologs (center), fragment predictions fall into one of three categories: no information, correct, or incorrect.
