# Supplementary material for: Knowledge-based Fragment Binding Prediction
Source: PLoS Comput Biol. 2014 Apr 24;10(4):e1003589. doi: 10.1371/journal.pcbi.1003589 (PMC3998881; doi:10.1371/journal.pcbi.1003589)
Supplement: Figure S9 — Sequence identity between test proteins and nearest neighbor proteins. (DOCX) [file pcbi.1003589.s009.docx]

**Figure S9. Sequence identity between test proteins and nearest neighbor proteins**


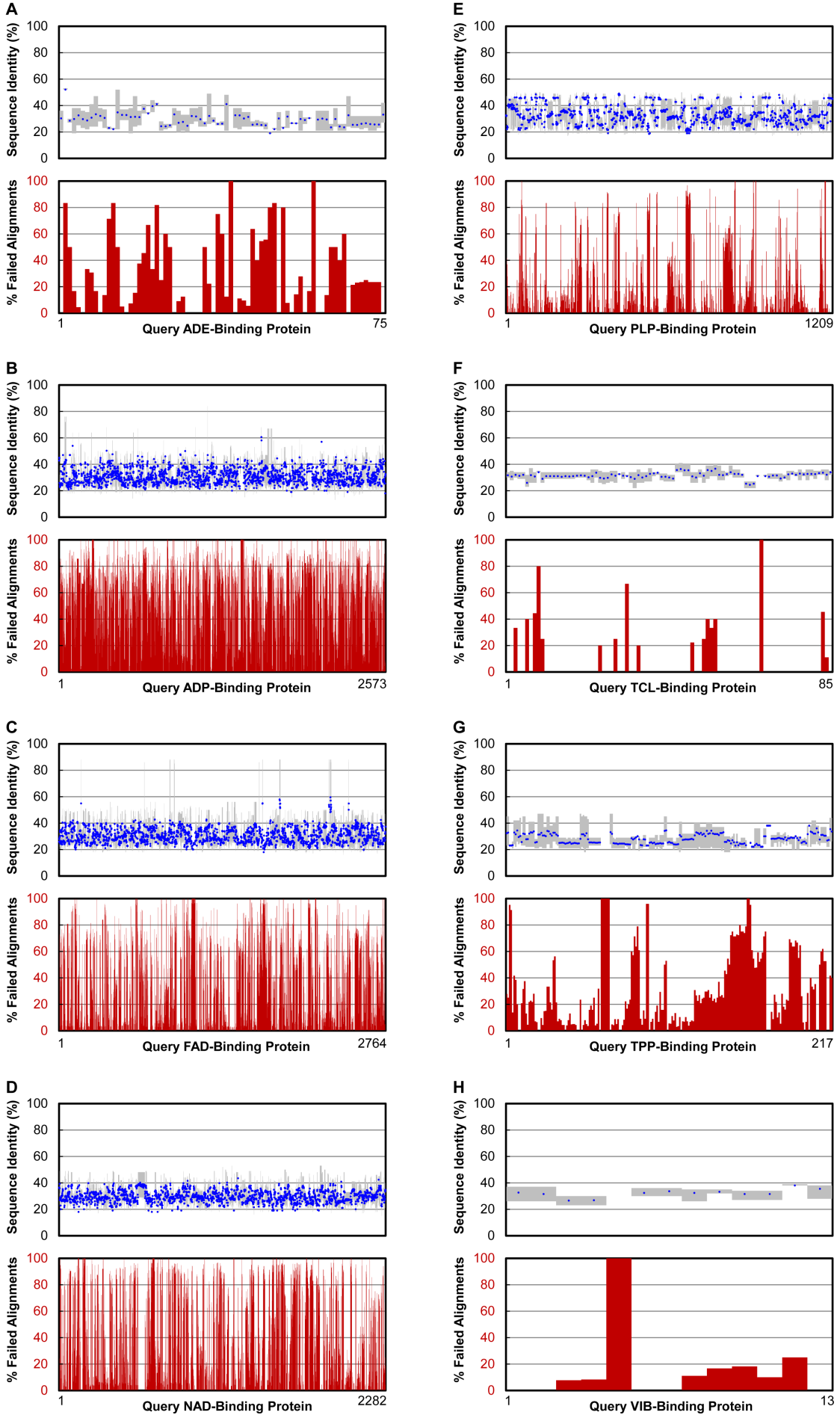


Each panel refers to a different validation ligand: (A) ADE (B) ADP (C) FAD (D) NAD (E) PLP (F) TCL

(G) TPP and (H) VIB with the x-axis representing query protein structures binding the validation ligand. Query proteins without a correct fragment prediction are not represented in the plots. The upper plot within each panel shows the sequence identity between query proteins and the nearest neighbor proteins used to make the most significant correct fragment prediction. The range of sequence identities observed between a query protein and its nearest neighbor proteins is represented as a vertical gray bar with a blue dot denoting the average. The lower plot within each panel shows the percentage of nearest neighbor proteins that fail to align to each query protein.
