# Supplementary material for: Knowledge-based Fragment Binding Prediction
Source: PLoS Comput Biol. 2014 Apr 24;10(4):e1003589. doi: 10.1371/journal.pcbi.1003589 (PMC3998881; doi:10.1371/journal.pcbi.1003589)
Supplement: Figure S10 — Comparison of observed ligand binding pockets and predicted pockets. (DOCX) [file pcbi.1003589.s010.docx]

**Figure S10. Comparison of observed ligand binding pockets and predicted pockets**


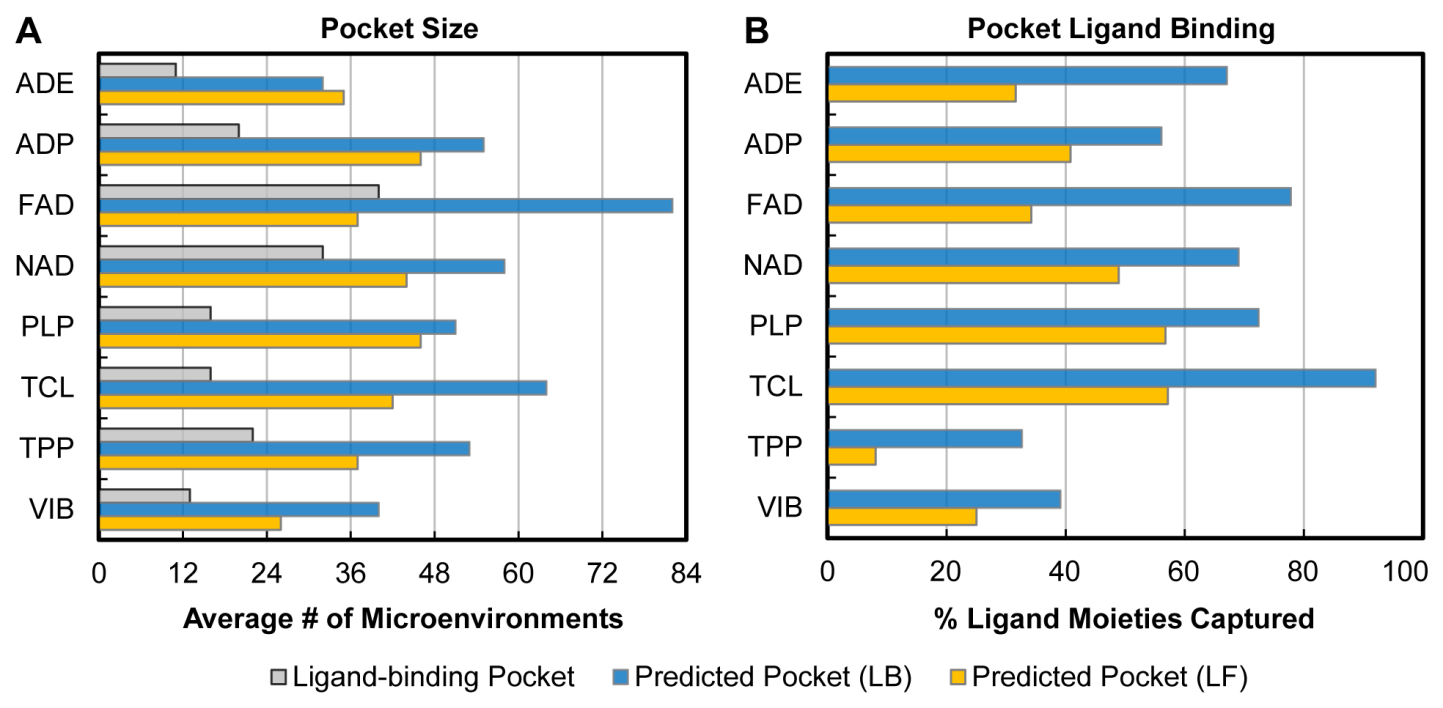


1. Pocket size comparison. Shown is the average number of microenvironments in the observed ligand-binding pockets and predicted pockets.
2. Pocket ligand binding comparison. Shown is the percent of ligand moieties bound by the observed ligand-binding pockets that are captured/found by the predicted pockets.

LB = ligand-bound structures

LF = ligand-free structures
