# Supplementary material for: Knowledge-based Fragment Binding Prediction
Source: PLoS Comput Biol. 2014 Apr 24;10(4):e1003589. doi: 10.1371/journal.pcbi.1003589 (PMC3998881; doi:10.1371/journal.pcbi.1003589)
Supplement: Figure S11 — FragFEATURE performance on predicted pockets. (DOCX) [file pcbi.1003589.s011.docx]

**Figure S11. FragFEATURE performance on predicted pockets**


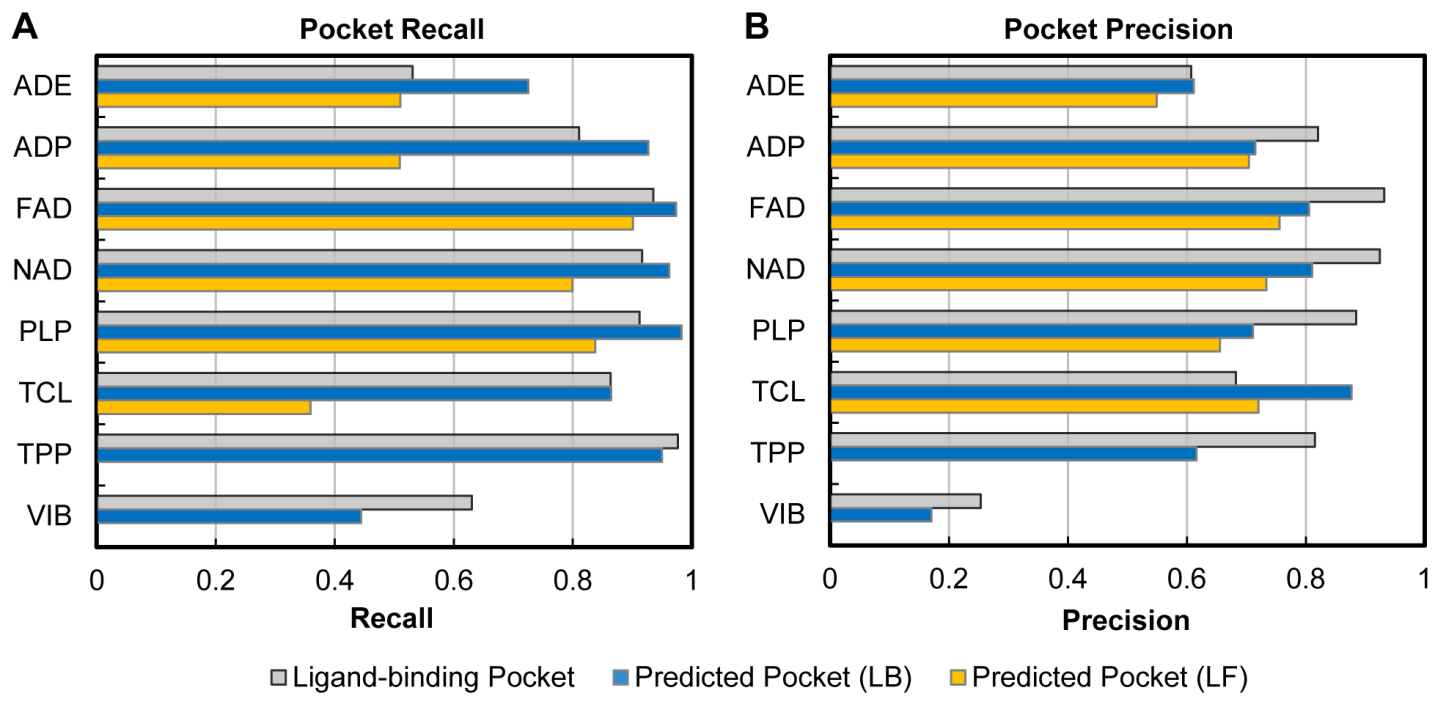


1. FragFEATURE recall on observed ligand-binding pockets and predicted pockets.
2. FragFEATURE precision on observed ligand-binding pockets and predicted pockets.

LB = ligand-bound structures

LF = ligand-free structures
