# Supplementary material for: Knowledge-based Fragment Binding Prediction
Source: PLoS Comput Biol. 2014 Apr 24;10(4):e1003589. doi: 10.1371/journal.pcbi.1003589 (PMC3998881; doi:10.1371/journal.pcbi.1003589)
Supplement: Figure S12 — Fragment prediction and validation for Abl tyrosine kinase. (DOCX) [file pcbi.1003589.s012.docx]

**Figure S12. Fragment prediction and validation for Abl tyrosine kinase**

**
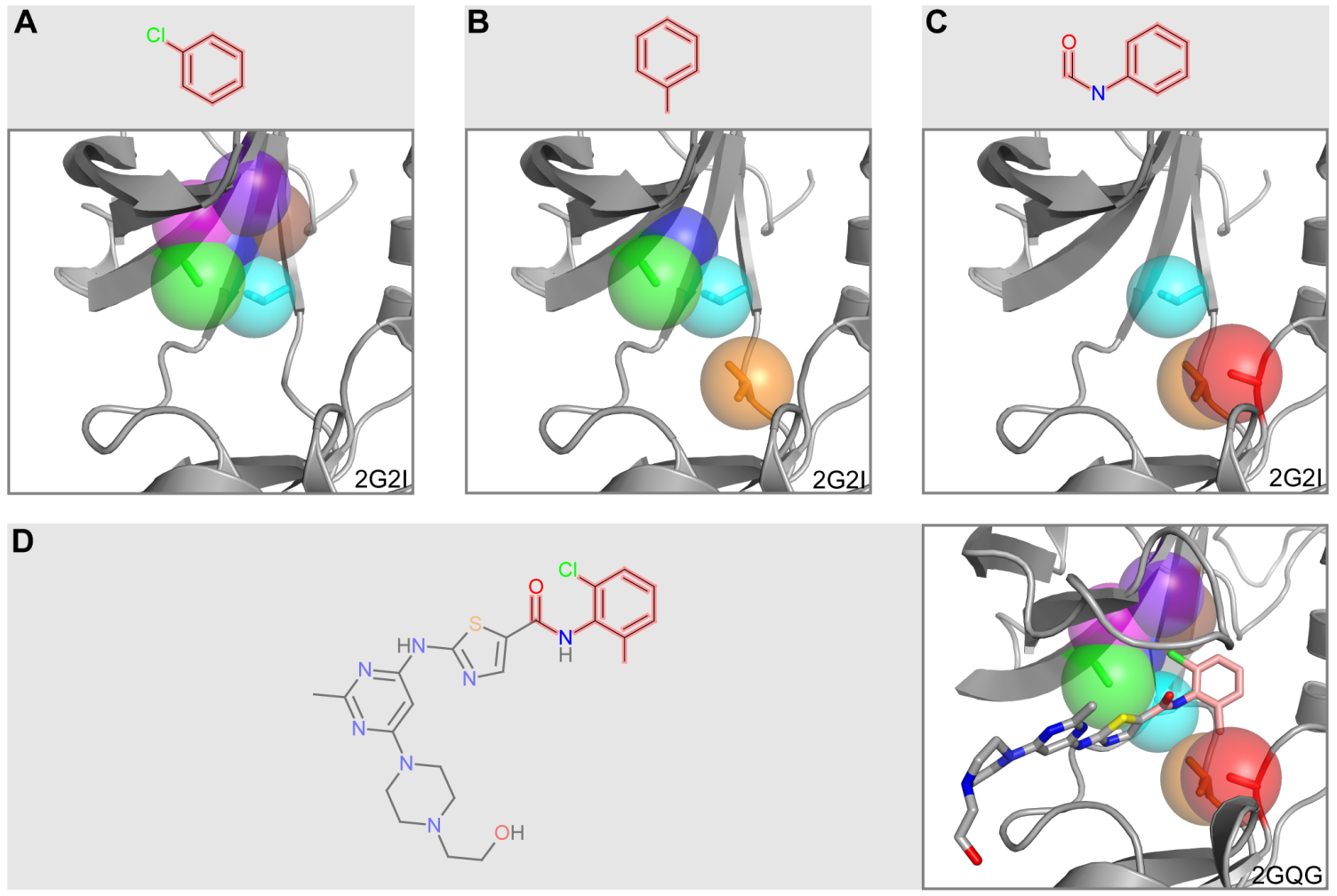
**

1. Fragment 7964 and the microenvironments from the query Abl kinase structure associated with the fragment prediction.
2. Fragment 1140 and the microenvironments from the query Abl kinase structure associated with the fragment prediction.
3. Fragment 7671 and the microenvironments from the query Abl kinase structure associated with the fragment prediction.
4. PDB ligand 1N1 (dasatinib) and an alternate structure of Abl kinase bound to 1N1. Fragment 7964, 1140 and 7671 substructures of 1N1 are in pink.

Proteins are shown in cartoon representation with microenvironments as semi-transparent spheres. Microenvironment color scheme is arbitrary but consistent between panels. Side chains corresponding to microenvironments are shown in stick representation. Ligands are also drawn in stick representation.
