# Supplementary material for: Knowledge-based Fragment Binding Prediction
Source: PLoS Comput Biol. 2014 Apr 24;10(4):e1003589. doi: 10.1371/journal.pcbi.1003589 (PMC3998881; doi:10.1371/journal.pcbi.1003589)
Supplement: Figure S13 — Fragment prediction and validation for protein kinase A (PKA). (DOCX) [file pcbi.1003589.s013.docx]

**Figure S13. Fragment prediction and validation for protein kinase A (PKA)**

**
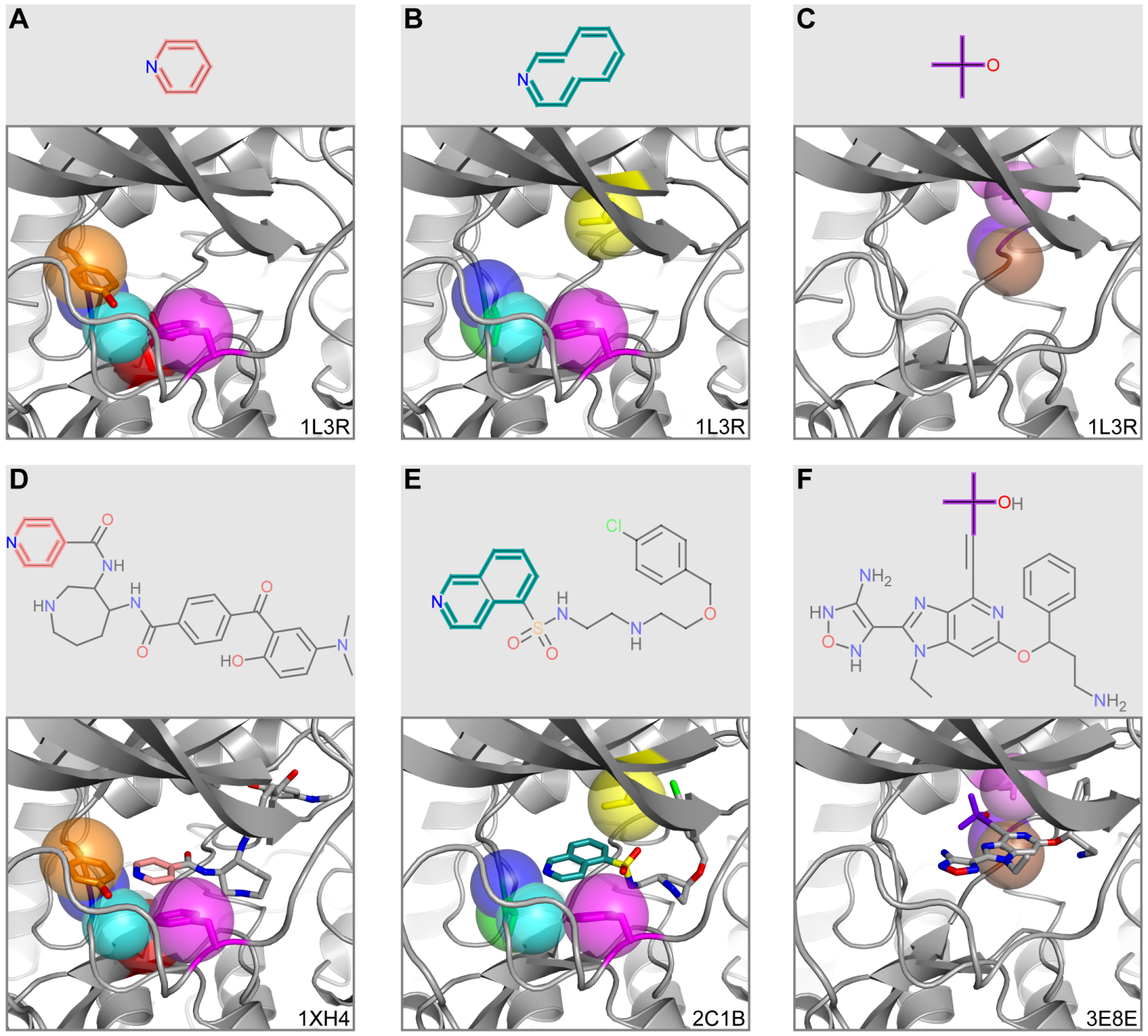
**

1. Fragment 1049 and the microenvironments from the query PKA structure associated with the fragment prediction.
2. Fragment 13287579 and the microenvironments from the query PKA structure associated with the fragment prediction.
3. Fragment 6386 and the microenvironments from the query PKA structure associated with the fragment prediction.
4. PDB ligand R69 and an alternate structure of PKA bound to R69. Fragment 1049 substructure of R69 is in pink.
5. PDB ligand CQP and an alternate structure of PKA bound to CQP. Fragment 13287579 substructure of CQP is in teal.
6. PDB ligand G98 and an alternate structure of PKA bound to G98. Fragment 6386 substructure of G98 is in purple.

Proteins are shown in cartoon representation with microenvironments as semi-transparent spheres. Microenvironment color scheme is arbitrary but consistent between panels. Side chains corresponding to microenvironments are shown in stick representation. Ligands are also drawn in stick representation.
