# Supplementary material for: Knowledge-based Fragment Binding Prediction
Source: PLoS Comput Biol. 2014 Apr 24;10(4):e1003589. doi: 10.1371/journal.pcbi.1003589 (PMC3998881; doi:10.1371/journal.pcbi.1003589)
Supplement: Figure S14 — Alternative fragments for PDB ligands I5S, M77, and TZ1. (DOCX) [file pcbi.1003589.s014.docx]

**Figure S14. Alternative fragments for PDB ligands I5S, M77, and TZ1**


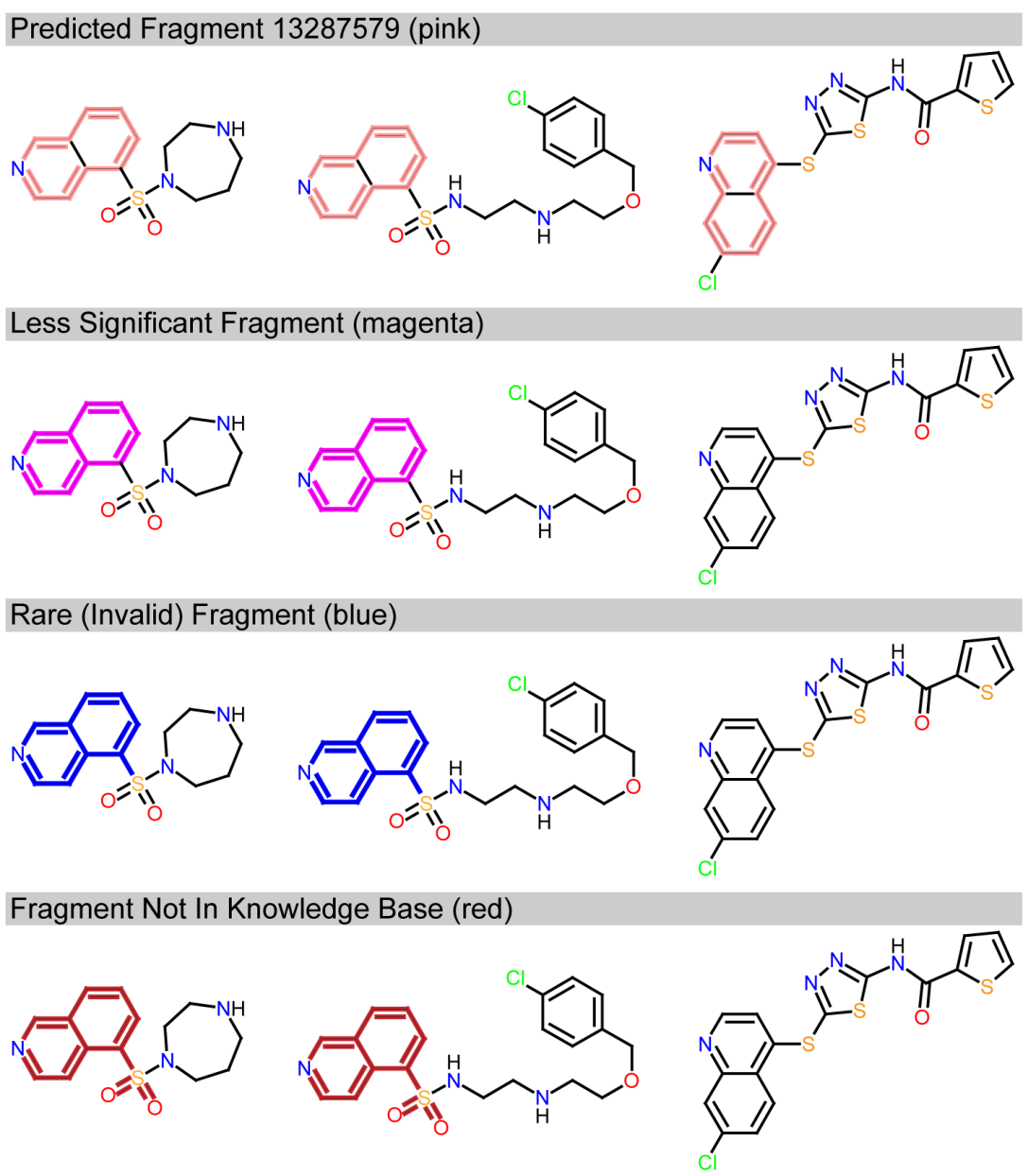


Multiple fragments are overlapping substructures of PDB ligands I5S (left), M77 (center), and TZ1 (right). The top row highlights the predicted fragment 13287579 (pink) with the following rows highlighting examples of reasonable fragments that are not predicted by FragFEATURE for various reasons.
