# Supplementary material for: Knowledge-based Fragment Binding Prediction
Source: PLoS Comput Biol. 2014 Apr 24;10(4):e1003589. doi: 10.1371/journal.pcbi.1003589 (PMC3998881; doi:10.1371/journal.pcbi.1003589)
Supplement: Figure S15 — Microenvironment similarity of nearest neighbors. (DOCX) [file pcbi.1003589.s015.docx]

**Figure S15. Microenvironment similarity of nearest neighbors**


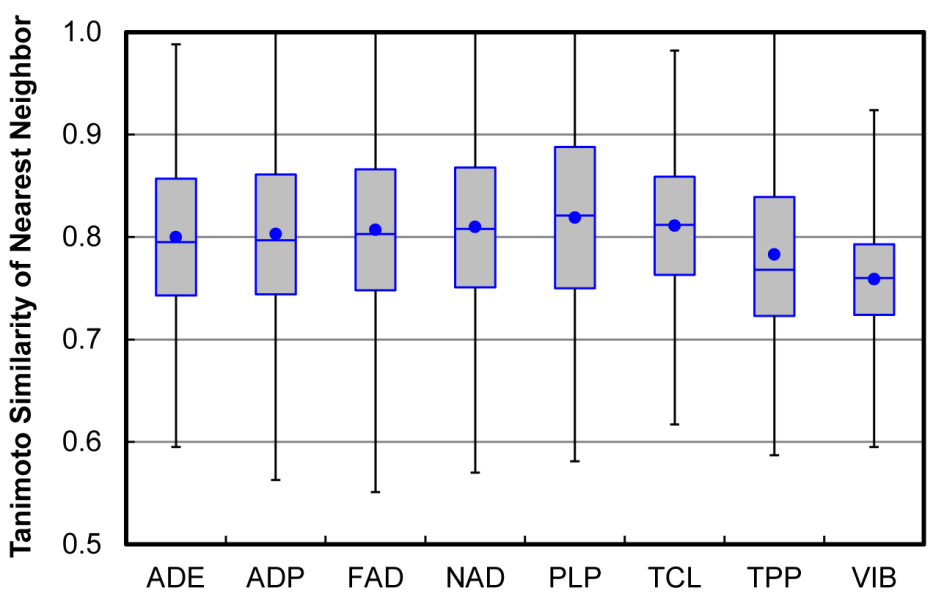


The boxplots summarize the distribution (min, first quartile, second quartile, third quartile, and max) of the Tanimoto similarity coefficient between microenvironments binding the validation ligands and their nearest knowledge base neighbor. Blue dots represent the mean. A Tanimoto similarity of 1.0 indicates identical microenvironments.
