# Supplementary figures and images for: Knowledge-based Fragment Binding Prediction
Source: PLoS Comput Biol. 2014 Apr 24;10(4):e1003589. doi: 10.1371/journal.pcbi.1003589 (PMC3998881; doi:10.1371/journal.pcbi.1003589)

**Figure S16. FragFEATURE computation time**


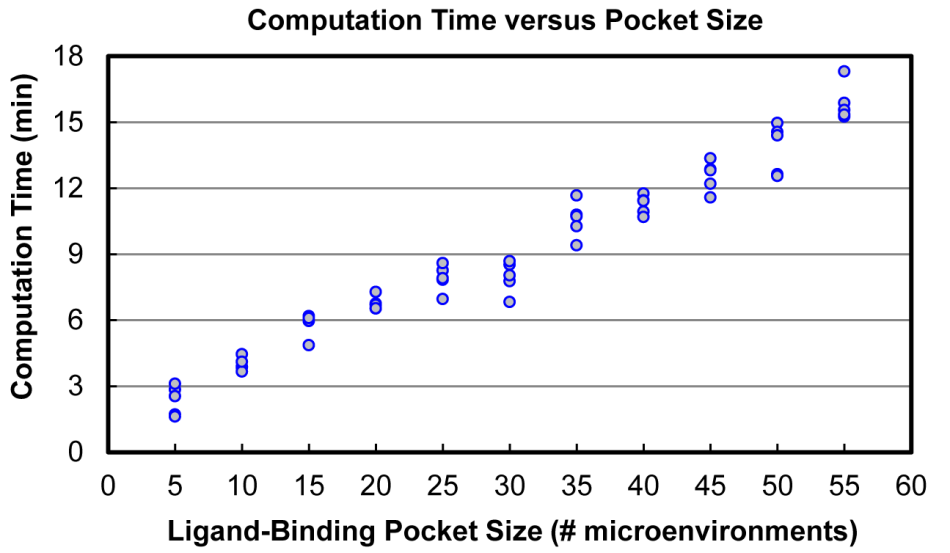

Supplement: Figure S16 — FragFEATURE computation time. (DOCX) [file pcbi.1003589.s016.docx]
