# Supplementary material for: Knowledge-based Fragment Binding Prediction
Source: PLoS Comput Biol. 2014 Apr 24;10(4):e1003589. doi: 10.1371/journal.pcbi.1003589 (PMC3998881; doi:10.1371/journal.pcbi.1003589)
Supplement: Table S1 — Prevalence of validation ligands across 50% sequence identity clusters. (DOCX) [file pcbi.1003589.s017.docx]

**Table S1. Prevalence of validation ligands across 50% sequence identity clusters**

| Validation Ligand | # 50% Sequence Identity Clusters |
| --- | --- |
| ADE | 55 |
| ADP | 573 |
| FAD | 336 |
| NAD | 303 |
| PLP | 185 |
| TCL | 6 |
| TPP | 36 |
| VIB | 10 |

Protein structures within the PDB have a pre-computed 50% sequence identity cluster ID. Proteins within a cluster possess at least 50% sequence identity with another cluster member. This table shows the number of cluster IDs possessing a member structure bound to the validation ligand.
