# Supplementary material for: Knowledge-based Fragment Binding Prediction
Source: PLoS Comput Biol. 2014 Apr 24;10(4):e1003589. doi: 10.1371/journal.pcbi.1003589 (PMC3998881; doi:10.1371/journal.pcbi.1003589)
Supplement: Table S2 — Breakdown of protein pockets tested. (DOCX) [file pcbi.1003589.s018.docx]

**Table S2. Breakdown of protein pockets tested**

| Validation Ligand | # Ligand-binding Pockets | # Predicted Pockets  (Ligand-bound structures) | # Predicted Pockets  (Ligand-free structures) |
| --- | --- | --- | --- |
| ADE | 123 | 111 | 475 |
| ADP | 2640 | 2452 | 3316 |
| FAD | 2769 | 2757 | 105 |
| NAD | 2309 | 2263 | 874 |
| PLP | 1227 | 1224 | 329 |
| TCL | 88 | 82 | 56 |
| TPP | 217 | 215 | 78 |
| VIB | 19 | 17 | 24 |
| Total | 9392 | 9121 | 5257 |
