# Supplementary material for: Knowledge-based Fragment Binding Prediction
Source: PLoS Comput Biol. 2014 Apr 24;10(4):e1003589. doi: 10.1371/journal.pcbi.1003589 (PMC3998881; doi:10.1371/journal.pcbi.1003589)
Supplement: Table S3 — PDB ligands supporting the benzamide prediction for exotoxin A. (DOCX) [file pcbi.1003589.s019.docx]

**Table S3. PDB ligands supporting the benzamide prediction for exotoxin A**

| **Ligand ID** | **Chemical Structure** | **# PDB**  **Occurrence** | **Ligand ID** | **Chemical Structure** | **# PDB**  **Occurrence** |
| --- | --- | --- | --- | --- | --- |
| 09L | 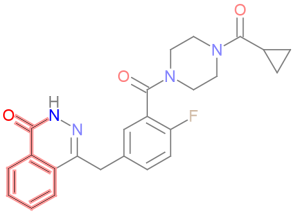 | 1 | DHQ | 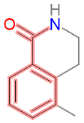 | 1 |
| 0RU | 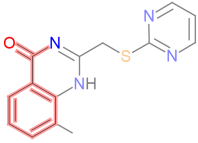 | 1 | FRM | 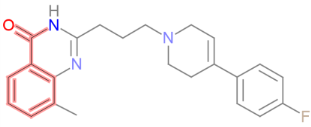 | 1 |
| 0RY | 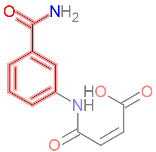 | 1 | FRQ | 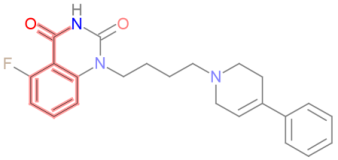 | 1 |
| 18N | 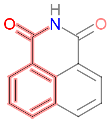 | 1 | G9D | 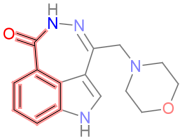 | 1 |
| 3AB | 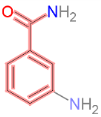 | 5 | G9G | 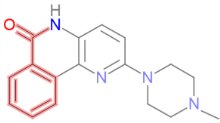 | 1 |
| 3GN | 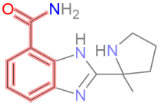 | 1 | G9H | 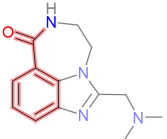 | 1 |
| 4AN | 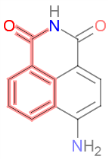 | 1 | G9L | 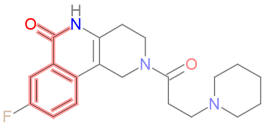 | 2 |
| 78P | 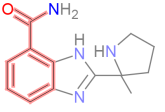 | 2 | KU8 | 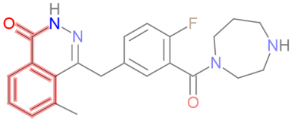 | 1 |
| BZC | 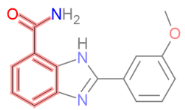 | 1 | P34 | 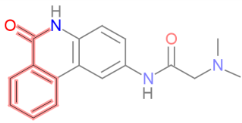 | 7 |
| CNQ | 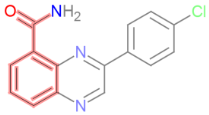 | 1 |  |  |  |

Benzamide substructure is shown in pink.
