# Supplementary material for: Knowledge-based Fragment Binding Prediction
Source: PLoS Comput Biol. 2014 Apr 24;10(4):e1003589. doi: 10.1371/journal.pcbi.1003589 (PMC3998881; doi:10.1371/journal.pcbi.1003589)
Supplement: Table S4 — PDB structures supporting the benzamide prediction for exotoxin A. (DOCX) [file pcbi.1003589.s020.docx]

**Table S4. PDB structures supporting the benzamide prediction for exotoxin A**

| **Microenvironment** | | **PDB ID of nearest neighbors that are binding benzamide** | | | | | | | |
| --- | --- | --- | --- | --- | --- | --- | --- | --- | --- |
| 3B78* | 1 | 1EFY* | 3C49* | 3KI0* | 3KCZ* |  |  |  |  |
| 3B78 | 2 |  | 3C49 |  |  | 4F1L* |  |  |  |
| 3B78 | 3 | 1UK1 |  | 3ESS |  |  | 3HKV* | 3U9Y* |  |
| 3B78 | 4 | 1UK0 |  | 2Q6M |  |  |  |  |  |
| 3B78 | 5 | 1PAX |  | 3KI3 | 3KCZ |  |  |  |  |
| 3B78 | 6 | 2RD6 |  | 3KI2 |  |  | 3HKV |  | 4F0D* |
| 3B78 | 7 |  |  | 3KI6 |  | 3GEY |  |  |  |
| 3B78 | 8 |  |  |  |  | 3GEY |  |  |  |
| 3B78 | 9 |  |  | 3KI0 |  |  |  |  |  |
| 3B78 | 10 | 2PAX |  | 2Q6M |  | 4F0E |  |  |  |
| **50% Sequence Identity Cluster ID** | | | | | | | | | |
| 1273 | | 1068 | 6144 | 4054 | 6872 | 1385 | 16529 | 522 | 15758 |

Columns 1 and 2 (shaded gray) represent exotoxin A and the ten microenvironments predicted to bind benzamide. The colored circles match the microenvironment colors in Figure 4. Columns 3-10 contain the nearest neighbor proteins retrieved by FragFEATURE that were contributing to the benzamide fragment prediction. Each row is thus an exotoxin A microenvironment and the subset of nearest neighbors binding benzamide. Proteins denoted with an asterisk were arbitrarily selected to calculate pairwise structural alignments and sequence identities using jFATCAT and DaliLite (Table S6). At the bottom of the table, each column is labeled by its 50% sequence identity cluster ID from the PDB. Each microenvironment has only one PDB ID per cluster ID because nearest neighbors are not permitted to share more than 50% sequence identity.
