# Supplementary material for: Knowledge-based Fragment Binding Prediction
Source: PLoS Comput Biol. 2014 Apr 24;10(4):e1003589. doi: 10.1371/journal.pcbi.1003589 (PMC3998881; doi:10.1371/journal.pcbi.1003589)
Supplement: Table S5 — Protein names of PDB structures supporting the benzamide prediction for exotoxin A. (DOCX) [file pcbi.1003589.s021.docx]

**Table S5. Protein names of PDB structures supporting the benzamide prediction for exotoxin A**

| **Protein Name** | **Species** | **50% Sequence Identity Cluster ID** |
| --- | --- | --- |
| Exotoxin A | *Pseudomonas aeruginosa* | 1273 |
| Poly [ADP-ribose] polymerase 1 | *Gallus gallus, Homo sapiens* | 1068 |
| Poly [ADP-ribose] polymerase 3 | *Homo sapiens* | 6144 |
| Cholix toxin | *Vibrio cholerae* | 4054 |
| Poly [ADP-ribose] polymerase 2 | *Homo sapiens* | 6872 |
| Poly [ADP-ribose] polymerase 14  Poly [ADP-ribose] polymerase 15 | *Homo sapiens* | 1385 |
| Poly [ADP-ribose] polymerase 10 | *Homo sapiens* | 16529 |
| Tankyrase-2 | *Homo sapiens* | 522 |
| Mono [ADP-ribose] polymerase 16 | *Homo sapiens* | 15758 |
