# Supplementary material for: Knowledge-based Fragment Binding Prediction
Source: PLoS Comput Biol. 2014 Apr 24;10(4):e1003589. doi: 10.1371/journal.pcbi.1003589 (PMC3998881; doi:10.1371/journal.pcbi.1003589)
Supplement: Table S6 — Sequence identity between PDB structures supporting the benzamide prediction for exotoxin A. (DOCX) [file pcbi.1003589.s022.docx]

**Table S6. Sequence identity between PDB structures supporting the benzamide prediction for exotoxin A**

| DaliLite  jFATCAT | | **50% Sequence Identity Cluster ID** | | | | | | | | |
| --- | --- | --- | --- | --- | --- | --- | --- | --- | --- | --- |
|  |  | 1273 | 1068 | 6144 | 4054 | 6872 | 1385 | 16529 | 522 | 15758 |
| **50% Sequence Identity Cluster ID** | 1273 |  | 15% | 13% | 41% | 15% | 12% | 17% | 14% | 15% |
|  | 1068 | 9.4% |  | 37% | 11% | 46% | 19% | 19% | 26% | 20% |
|  | 6144 | 8.3% | 34.4% |  | 18% | 37% | 25% | 21% | 27% | 14% |
|  | 4054 | 38.2% | 5.8% | 8.1% |  | 11% | 14% | 16% | 14% | 8% |
|  | 6872 | 8.4% | 45.8% | 34.3% | 6.2% |  | 15% | 17% | 29% | 18% |
|  | 1385 | 8.8% | 13.5% | 18.6% | 7.1% | 10.1% |  | 38% | 29% | 16% |
|  | 16529 | 7.0% | 13.2% | 13.5% | 11.6% | 11.8% | 37.6% |  | 24% | 15% |
|  | 522 | 11.2% | 19.0% | 19.6% | 7.6% | 21.2% | 22.1% | 19.4% |  | 17% |
|  | 15758 | 7.8% | 11.4% | 9.0% | 4.6% | 10.7% | 10.7% | 8.6% | 9.1% |  |

Table values represent pairwise percent sequence identity calculated from a structural alignment of proteins using jFATCAT (lower triangle) or DaliLite (upper triangle). For the PDB IDs of the proteins used, refer to Table S4. The row and column highlighted gray correspond to the 50% sequence identity cluster ID for exotoxin A.
