# Supplementary material for: Knowledge-based Fragment Binding Prediction
Source: PLoS Comput Biol. 2014 Apr 24;10(4):e1003589. doi: 10.1371/journal.pcbi.1003589 (PMC3998881; doi:10.1371/journal.pcbi.1003589)
Supplement: Table S7 — PDB structures supporting fragment 13509097/benzamide prediction for DAPK1. (DOCX) [file pcbi.1003589.s023.docx]

**Table S7. PDB structures supporting fragment 13509097/benzamide prediction for DAPK1**

| **Protein Name** | **Kinase Type** | **Species** | **50% Sequence Identity Cluster ID** | **PDB ID** |
| --- | --- | --- | --- | --- |
| Death-associated protein kinase 1 | serine/threonine | *Homo sapiens* | 375 | 2W4J* |
| Dual specificity mitogen-activated protein kinase kinase 6 | dual specificity | *Homo sapiens* | 8257 | 3FME* |
| Tyrosine-protein kinase Fes/Fps | tyrosine | *Homo sapiens* | 9420 | 3CD3* |
| Dual specificity mitogen-activated protein kinase kinase 1 | dual specificity | *Homo sapiens* | 1004 | 3EQF* |
| Protein kinase C theta type | serine/threonine | *Homo sapiens* | 2292 | 1XJD* |
| Angiopoietin-1 receptor | tyrosine | *Homo sapiens* | 5335 | 3L8P* |
| Ribosomal protein S6 kinase beta-1 | serine/threonine | *Homo sapiens* | 2175 | 3A60* |
| Calcium/calmodulin-dependent protein kinase kinase 2 | serine/threonine | *Homo sapiens* | 21519 | 2ZV2* |
| cAMP-dependent protein kinase catalytic subunit alpha | serine/threonine | *Homo sapiens* | 135 | 3AMA* |

Column 5 refers to the specific PDB structures used by FragFEATURE to make the fragment predictions. The highlighted row corresponds to the query DAPK1 protein. Proteins denoted with an asterisk were used to calculate pairwise structural alignments and sequence identities using DaliLite and jFATCAT (Table S8).
