# Supplementary material for: Knowledge-based Fragment Binding Prediction
Source: PLoS Comput Biol. 2014 Apr 24;10(4):e1003589. doi: 10.1371/journal.pcbi.1003589 (PMC3998881; doi:10.1371/journal.pcbi.1003589)
Supplement: Table S8 — Sequence identity of PDB structures supporting fragment 13509097/benzamide prediction for DAPK1. (DOCX) [file pcbi.1003589.s024.docx]

**Table S8. Sequence identity of PDB structures supporting fragment 13509097/benzamide prediction for DAPK1**

| **50% Sequence Identity Cluster ID** | **Percent Sequence Identity to DAPK1 (Cluster ID: 375)**  **DaliLite / jFATCAT** |
| --- | --- |
| 8257 | 25% / 21.9% |
| 9420 | 24% / 22.1% |
| 1004 | 26% / 23.0% |
| 2292 | 29% / 27.3% |
| 5335 | 24% / 20.1% |
| 2175 | 29% / 25.4% |
| 21519 | 31% / 27.5% |
| 135 | 31% / 27.4% |

Table values represent pairwise percent sequence identity calculated from a structural alignment of nearest neighbor proteins to the query DAPK1 protein using DaliLite or jFATCAT. For the PDB IDs of the proteins used, refer to Table S7.
