# Supplementary material for: Knowledge-based Fragment Binding Prediction
Source: PLoS Comput Biol. 2014 Apr 24;10(4):e1003589. doi: 10.1371/journal.pcbi.1003589 (PMC3998881; doi:10.1371/journal.pcbi.1003589)
Supplement: Table S9 — PDB ligands supporting the benzamide prediction for DAPK1. (DOCX) [file pcbi.1003589.s025.docx]

**Table S9. PDB ligands supporting the benzamide prediction for DAPK1**

| **Ligand ID** | **Chemical Structure** | **# PDB**  **Occurrence** | **Ligand ID** | **Chemical Structure** | **# PDB**  **Occurrence** |
| --- | --- | --- | --- | --- | --- |
| 0CE | 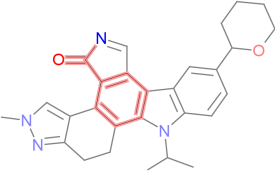 | 1 | SKE | 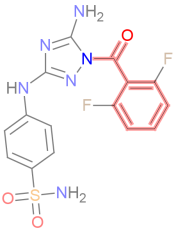 | 1 |
| 609 | 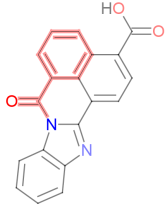 | 1 | STU | 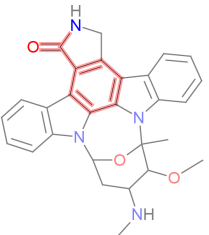 | 46 |
| KSA | 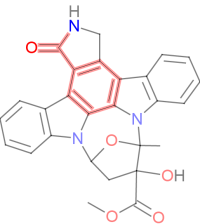 | 3 |  |  |  |

Benzamide substructure is shown in pink.
