# Supplementary material for: Knowledge-based Fragment Binding Prediction
Source: PLoS Comput Biol. 2014 Apr 24;10(4):e1003589. doi: 10.1371/journal.pcbi.1003589 (PMC3998881; doi:10.1371/journal.pcbi.1003589)
Supplement: Table S10 — PDB structures supporting fragment 1049/241 prediction for aPKC. (DOCX) [file pcbi.1003589.s026.docx]

**Table S10. PDB structures supporting fragment 1049/241 prediction for aPKC**

| **Protein Name** | **Kinase Type** | **Species** | **50% Sequence Identity Cluster ID** | **PDB ID(s)** |
| --- | --- | --- | --- | --- |
| Protein kinase C iota type | serine/threonine | *Mus musculus* | 2295 | 4DC2* |
| cAMP-dependent protein kinase catalytic subunit alpha | serine/threonine | *Bos Taurus*, *Homo sapiens* | 135 | 2GNL*, 3VQH, 2GNJ, 3NX8 |
| Glycogen synthase kinase-3 beta | serine/threonine | *Homo sapiens* | 408 | 4DIT*, 1Q4L |
| Rho-associated protein kinase 1 | serine/threonine | *Homo sapiens* | 1070 | 2ETR* |
| Serine/threonine-protein kinase MRCK beta | serine/threonine | *Homo sapiens* | 6111 | 3TKU* |
| Non-receptor tyrosine-protein kinase TYK2 | tyrosine | *Homo sapiens* | 214 | 3NYX* |
| RAC-beta serine/threonine-protein kinase | serine/threonine | *Homo sapiens* | 1071 | 3E87* |
| Proto-oncogene tyrosine-protein kinase Src | tyrosine | *Gallus gallus* | 168 | 3G6H* |
| UDP-glucose 4-epimerase | N/A | *Bacillus anthracis* | 6978 | 2C20* |
| Beta-secretase 1 | N/A | *Homo sapiens* | 20 | 3IXK* |
| 3-hydroxy-3-methylglutaryl-coenzyme A reductase | N/A | *Homo sapiens* | 361 | 3CDB* |
| Ribosomal protein S6 kinase alpha-1 | serine/threonine | *Homo sapiens* | 2177 | 2Z7R* |
| MAP kinase-activated protein kinase 2 | serine/threonine | *Homo sapiens* | 376 | 3KC3* |
| Receptor tyrosine-protein kinase erbB-4 | tyrosine | *Homo sapiens* | 289 | 2R4B* |

Column 5 refers to the specific PDB structures used by FragFEATURE to make the fragment predictions. The highlighted row corresponds to the query aPKC protein. Proteins denoted with an asterisk are used to calculate pairwise structural alignments and sequence identities using DaliLite and jFATCAT (Table S11). Kinase type of N/A indicates the protein is not part of the protein kinase superfamily.
