# Supplementary material for: Knowledge-based Fragment Binding Prediction
Source: PLoS Comput Biol. 2014 Apr 24;10(4):e1003589. doi: 10.1371/journal.pcbi.1003589 (PMC3998881; doi:10.1371/journal.pcbi.1003589)
Supplement: Table S11 — Sequence identity of PDB structures supporting fragment 1049/241 prediction for aPKC. (DOCX) [file pcbi.1003589.s027.docx]

**Table S11. Sequence identity of PDB structures supporting fragment 1049/241 prediction for aPKC**

| **50% Sequence Identity Cluster ID** | **Percent Sequence Identity to aPKC (Cluster ID: 2295)**  **DaliLite / jFATCAT** |
| --- | --- |
| 135 | 38% / 35.7% |
| 408 | 25% / 20.7% |
| 1070 | 36% / 32.9% |
| 6111 | 36% / 30.7% |
| 214 | 21% / 16.9% |
| 1071 | 48% / 45.3% |
| 168 | 20% / 18% |
| 6978 | Failed to align / 3% |
| 20 | Failed to align / 3% |
| 361 | 3% / 4.4% |
| 2177 | 42% / 37.8% |
| 376 | 27% / 21.6% |
| 289 | 20% / 16.9% |

Table values represent pairwise percent sequence identity calculated from a structural alignment of nearest neighbor proteins to the query aPKC protein using DaliLite or jFATCAT. For the PDB IDs of the proteins used, refer to Table S10.
