# Supplementary material for: Knowledge-based Fragment Binding Prediction
Source: PLoS Comput Biol. 2014 Apr 24;10(4):e1003589. doi: 10.1371/journal.pcbi.1003589 (PMC3998881; doi:10.1371/journal.pcbi.1003589)
Supplement: Text S1 — Similar microenvironments bind similar fragments. (DOCX) [file pcbi.1003589.s028.docx]

**Text S1. Similar microenvironments bind similar fragments**

The utility of the knowledge base lies in the assumption that similar microenvironments will prefer to bind similar fragments. We validate this by generating fragment predictions for each microenvironment in the non-redundant knowledge base using fragment information from the nearest neighbor microenvironments (see *Performance of Fragment Prediction for Single Microenvironments*). We find a microenvironment tends to bind the same fragments as its nearest neighbors, often with the most significant predicted fragment matching the ligand bound. Using these predictions, we calculate a precision recall curve for each microenvironment type and measure performance using the area under the precision recall curve (AUPR). An AUPR approaching 1.0 indicates correct fragment predictions for each microenvironment. We observe reasonable performance with an average AUPR of 0.64 (Figure S5C). Thus, protein regions of similar physicochemical properties as captured by microenvironments prefer to bind similar fragments.

However, this performance varies with two parameters: *k* defines the number of nearest neighbor microenvironments to use and *M* restricts predicted fragments to those with sufficient prevalence in the knowledge base (see *Fragment Prediction for Single Microenvironments*). Parameter variation changes fragment hypergeometric p-values and thus fragment prediction performance. We tested a range of parameter values to capture the change in performance, with *k* = [1, 5, 10, 15, 20] and *M* ≥ [1, 5, 10, 15, 20, 25, 50, 100]. We observed the performance as measured by the AUPR to plateau as *k* approached 20 and *M* approached 100 (Figure S5A). While there is good performance for the larger values of *k* and *M*, we did not select these values. Large *k* and/or *M* favor prediction of common fragments (e.g. fragments bound by a large number of microenvironments) (Figure S5B). This would limit FragFEATURE’s ability to predict drug-like fragments, which do not necessarily have high prevalence in the knowledge base. To avoid this prediction bias, we set *k* = 5 and *M* ≥ 5. We do not use smaller values (i.e. *k* = 1 and *M* ≥ 1) because a smaller *k* relies too strongly on the similarity metric to perfectly rank the microenvironments, while a smaller *M* allows prediction of fragments whose microenvironment preferences are poorly understood. Thus, *k* and *M* are not set to values giving the best performance as measured by the AUPR but rather to values giving unbiased predictions.
