# Supplementary material for: Knowledge-based Fragment Binding Prediction
Source: PLoS Comput Biol. 2014 Apr 24;10(4):e1003589. doi: 10.1371/journal.pcbi.1003589 (PMC3998881; doi:10.1371/journal.pcbi.1003589)
Supplement: Text S2 — FragFEATURE predicts fragments of bound ligands using predicted protein pockets. (DOCX) [file pcbi.1003589.s029.docx]

**Text S2. FragFEATURE predicts fragments of bound ligands using predicted protein pockets**

In real use cases of FragFEATURE, a protein’s ligand-binding pocket may be poorly defined or unknown. We simulated this scenario by using a pocket-finding algorithm (fPocket) to identify pockets from the ligand-bound structures for analysis. fPocket performed pocket detection on each protein chain individually with ligand information removed. We analyzed the largest pocket of each protein chain as most small molecules tend to bind proteins in their largest pocket [[1](#_ENREF_1)] or largest predicted pocket [[2](#_ENREF_2)]. While the predicted pockets are much larger than the observed ligand-binding pockets (Figure S10A), they do not always rediscover the ligand-binding site. The predicted pockets bind only 33% to 92% of the ligand moieties bound by the observed pockets (Figure S10B). The predicted pockets are thus non-ideal because they miss protein regions involved in ligand binding and include extraneous protein regions not involved in ligand binding.

FragFEATURE predicted fragments for 9,121 pockets from the ligand-bound protein structures (Table S2). A large number of predictions corresponded to regions of the protein with no ligand-binding information in the analyzed structure or homolog structures. We could not evaluate the validity of these predictions and thus excluded them. On average, predicted pockets compared to ideal ligand-binding pockets showed consistent recall (85% versus 82%) (Figure S11A) and diminished precision (66% versus 74%) (Figure S11B). This indicates missing ligand-binding microenvironments do not have a significant impact while the extraneous microenvironments introduce noise (decreased precision). Ligand moieties not bound by the predicted pockets do not factor into the recall calculation. As the changes are moderate, FragFEATURE demonstrates resiliency to non-ideal pocket definitions.

**References**

1. Laskowski RA, Luscombe NM, Swindells MB, Thornton JM (1996) Protein clefts in molecular recognition and function. Protein Sci 5: 2438-2452.

2. An J, Totrov M, Abagyan R (2005) Pocketome via comprehensive identification and classification of ligand binding envelopes. Mol Cell Proteomics 4: 752-761.
