# Supplementary material for: Knowledge-based Fragment Binding Prediction
Source: PLoS Comput Biol. 2014 Apr 24;10(4):e1003589. doi: 10.1371/journal.pcbi.1003589 (PMC3998881; doi:10.1371/journal.pcbi.1003589)
Supplement: Text S3 — FragFEATURE predicts fragments of unbound ligands using predicted protein pockets. (DOCX) [file pcbi.1003589.s030.docx]

**Text S3. FragFEATURE predicts fragments of unbound ligands using predicted protein pockets**

Changes to pocket conformation, such as upon ligand binding, can alter pocket definition as well as pocket microenvironments. We therefore applied FragFEATURE to predicted pockets from ligand-free structures that possessed a homolog counterpart bound to a validation ligand. The largest pocket returned by fPocket remained larger than the observed ligand-binding pocket (Figure S10A). However, fPocket on the ligand-free structures compared to the ligand-bound structures showed diminished ability to find the ligand-binding site. The predicted pocket microenvironments when mapped to their counterpart ligand-bound structures were in contact with only a fraction of the ligand moieties bound (Figure S10B) (see *FragFEATURE Recall and Precision*). The ligand state of a protein structure therefore affects pocket finding even though ligand information is not used in this process. For PDB ligands TPP and VIB, the predicted pockets found less than 25% of the ligand moieties bound by the ligand-bound structures. We thus focused on ligand-free structures homologous to proteins binding ADE, ADP, FAD, NAD, PLP, and TCL as fPocket retrieved pockets more relevant for analysis.

FragFEATURE predicted fragments for 5,155 pockets from ligand-free protein structures (Table S2). We again excluded fragment predictions in regions of the protein with no available ligand-binding information. Compared to FragFEATURE performance on ideal ligand-binding pockets, recall and precision both decreased. On average, recall of ADE, ADP, FAD, NAD, PLP, and TCL moieties decreased moderately (65% versus 83%) (Figure S11A). Here, recall only included ligand moieties bound by the microenvironments of the predicted pockets in the counterpart ligand-bound structures. Precision also decreased moderately (69% versus 81%) (Figure S11B). FragFEATURE as implemented thus shows moderate ability to manage missing and extraneous microenvironments as well as changes to the microenvironments themselves due to altered pocket conformation. The structures tested here reflect likely use cases of FragFEATURE where no ligand is bound and prior information about the binding site is unavailable.
