# Supplementary material for: Knowledge-based Fragment Binding Prediction
Source: PLoS Comput Biol. 2014 Apr 24;10(4):e1003589. doi: 10.1371/journal.pcbi.1003589 (PMC3998881; doi:10.1371/journal.pcbi.1003589)
Supplement: Text S4 — Inhibitor fragment predictions for cholix toxin. (DOCX) [file pcbi.1003589.s031.docx]

**Text S4. Inhibitor fragment predictions for cholix toxin**

*Vibrio cholerae* is a Gram-negative bacterium that can cause cholera in humans. It secretes a variety of virulence factors including cholix toxin. This toxin inhibits eukaryotic ribosomal elongation factor 2 like exotoxin A, though the two share only 37% sequence identity [[1](#_ENREF_1)]. We analyzed cholix toxin bound to its natural ligand NAD (PDB ID: 3Q9O [[2](#_ENREF_2)]) and found results similar to those for exotoxin A. Microenvironments in proximity to the nicotinamide strongly predict fragment 2331 (benzamide) with a p-value of 7.8 x 10^-17^. Eleven microenvironments contribute to this fragment prediction and their nearest neighbors are bound to ligands containing fragment 2331 as a substructure, including 0RU, 3AB, 3GN, 4AN, BZC, CNQ, DHQ, KU8, and P34 (Table S3). Already available is a crystal structure of the catalytic fragment of cholix toxin in complex with P34 (PDB ID: 2Q6M [[3](#_ENREF_3)]), a competitive inhibitor (K_D_: 510nM). As the predicted fragment 2331 is a substructure of P34, it is a suitable fragment starting point for drug discovery for cholix toxin.

**References**

1. Purdy AE, Balch D, Lizarraga-Partida ML, Islam MS, Martinez-Urtaza J, et al. (2010) Diversity and distribution of cholix toxin, a novel ADP-ribosylating factor from Vibrio cholerae. Environ Microbiol Rep 2: 198-207.

2. Jorgensen R, Purdy AE, Fieldhouse RJ, Kimber MS, Bartlett DH, et al. (2008) Cholix toxin, a novel ADP-ribosylating factor from Vibrio cholerae. J Biol Chem 283: 10671-10678.

3. Fieldhouse RJ, Jorgensen R, Lugo MR, Merrill AR (2012) The 1.8 A cholix toxin crystal structure in complex with NAD+ and evidence for a new kinetic model. J Biol Chem 287: 21176-21188.
