# Supplementary material for: Knowledge-based Fragment Binding Prediction
Source: PLoS Comput Biol. 2014 Apr 24;10(4):e1003589. doi: 10.1371/journal.pcbi.1003589 (PMC3998881; doi:10.1371/journal.pcbi.1003589)
Supplement: Text S5 — Inhibitor fragment predictions for Abl tyrosine kinase. (DOCX) [file pcbi.1003589.s032.docx]

**Text S5. Inhibitor fragment predictions for Abl tyrosine kinase**

Abl tyrosine kinase is a well-studied protein kinase whose improper activation leads to chronic myeloid leukemia (CML) [[1](#_ENREF_1)]. Inhibition of Abl using Imatinib is a primary therapy but resistance is emerging [[2](#_ENREF_2)]. There is therefore significant interest in developing new inhibitors for the Abl kinase domain. FragFEATURE on the kinase domain bound to ADP (PDB ID: 2G2I [[3](#_ENREF_3)]) predicted three statistically significant fragments for three overlapping microenvironment sets to the right of the nucleotide-binding site. The first set from residues Ala269, Val270, Lys271, Ile313, and Thr315 predicted fragment 7964 with a p-value of 2.3 x 10^-10^ (Figure S12A). The second set from residues Ala269, Val299, and Thr315 predicted fragment 1140 with a p-value of 1.3 x 10^-9^ (Figure S12B). The third set from residues Val299, Thr315, and Ala380 predicted fragment 7671 with a p-value of 4.6 x 10^-9^ (Figure S12C).

These microenvironment sets occupy a similar spatial space, indicating the predicted fragments are mutually exclusive or originated from a common core scaffold. The latter is probable given all fragment predictions include a benzene ring. We validated these fragments with an alternate structure of Abl kinase bound to dasatinib (PDB ID: 2GQG [[4](#_ENREF_4)]), a potent inhibitor with sub-nanomolar binding affinity. All three fragments are overlapping substructures of dasatinib and are surrounded by the microenvironments predicting them (Figure S12D). These fragments thus also demonstrate non-independent fragment predictions, where the fragments collectively suggest a larger aggregate molecule (chemical structure) that is correlated with bioactivity against the protein target.

**References**

1. An X, Tiwari AK, Sun Y, Ding PR, Ashby CR, Jr., et al. (2010) BCR-ABL tyrosine kinase inhibitors in the treatment of Philadelphia chromosome positive chronic myeloid leukemia: a review. Leuk Res 34: 1255-1268.

2. Druker BJ, Tamura S, Buchdunger E, Ohno S, Segal GM, et al. (1996) Effects of a selective inhibitor of the Abl tyrosine kinase on the growth of Bcr-Abl positive cells. Nat Med 2: 561-566.

3. Levinson NM, Kuchment O, Shen K, Young MA, Koldobskiy M, et al. (2006) A Src-like inactive conformation in the abl tyrosine kinase domain. PLoS Biol 4: e144.

4. Tokarski JS, Newitt JA, Chang CY, Cheng JD, Wittekind M, et al. (2006) The structure of Dasatinib (BMS-354825) bound to activated ABL kinase domain elucidates its inhibitory activity against imatinib-resistant ABL mutants. Cancer Res 66: 5790-5797.
