# Supplementary material for: Knowledge-based Fragment Binding Prediction
Source: PLoS Comput Biol. 2014 Apr 24;10(4):e1003589. doi: 10.1371/journal.pcbi.1003589 (PMC3998881; doi:10.1371/journal.pcbi.1003589)
Supplement: Text S6 — Inhibitor fragment predictions for protein kinase A. (DOCX) [file pcbi.1003589.s033.docx]

**Text S6. Inhibitor fragment predictions for protein kinase A**

Hormonal and extracellular signals alter cellular cyclic AMP levels, affecting the behavior of downstream effectors like protein kinase A (PKA) [[1](#_ENREF_1)]. PKA regulates numerous processes including ion channel permeability, gene expression, and cell proliferation. Given the catalytic subunit of ADP-bound PKA (PDB ID: 1L3R [[2](#_ENREF_2)]), FragFEATURE predicts multiple fragments unrelated to the native ligand. Proximal to the adenine-binding site, FragFEATURE predicts fragment 1049 with a p-value of 1.9 x 10^-11^ using five microenvironments centered on residues Tyr122, Val123, Leu173, and Phe327 (Figure S13A). Already available is PKA bound to PDB ligand R69 (PDB ID: 1XH4 [[3](#_ENREF_3)]), a potent PKA inhibitor (IC50: 30nM). R69 possesses fragment 1049 as a substructure that in the inhibitor-bound structure is surrounded by the microenvironments predicting it (Figure S13D).

For two additional microenvironment sets from residues Val57, Val123, and Phe327, FragFEATURE predicts fragment 13287579 with a p-value of 2.2 x 10^-8^ (Figure S13B) using information from PDB ligands I5S, M77, and TZ1. Examination of these ligands reveals alternative fragments that FragFEATURE does not report because the fragment is less significant, is too rare and excluded from prediction, or is not a knowledge base fragment (Figure S14). Visual inspection of the ligands supporting a prediction can thus lead to interesting fragment insights. We identified another inhibitor-bound PKA structure (PDB ID: 2C1B [[4](#_ENREF_4)]) where the inhibitor, CQP (IC50: 400nM), possesses fragment 13287579 as a substructure. This fragment substructure is surrounded by the corresponding microenvironments of 2C1B (Figure S13E).

FragFEATURE also predicts fragment 6386 with a p-value of 2.2 x 10^-4^ for a spatially removed microenvironment set from residues Glu91, Asp184, and Phe185 (Figure S13C). An existing structure of PKA in complex with inhibitor G98 (PDB ID: 3E8E [[5](#_ENREF_5)]) structurally validates the fragment, as it is a substructure of G98 and is in proximity to the microenvironments that predict it (Figure S13F). The predicted fragments thus each correspond to a unique PKA inhibitor. This case study highlights FragFEATURE’s ability to predict multiple fragments for a protein pocket that can serve as independent starting points for drug discovery. Interestingly, no compounds active against PKA possess fragment 1049/11182733 with fragment 6386. As they occupy different regions of the pocket, linkage of the fragments into a new inhibitor is theoretically feasible.

**References**

1. Edwards HV, Christian F, Baillie GS (2012) cAMP: novel concepts in compartmentalised signalling. Semin Cell Dev Biol 23: 181-190.

2. Madhusudan, Akamine P, Xuong NH, Taylor SS (2002) Crystal structure of a transition state mimic of the catalytic subunit of cAMP-dependent protein kinase. Nat Struct Biol 9: 273-277.

3. Breitenlechner CB, Friebe WG, Brunet E, Werner G, Graul K, et al. (2005) Design and crystal structures of protein kinase B-selective inhibitors in complex with protein kinase A and mutants. J Med Chem 48: 163-170.

4. Collins I, Caldwell J, Fonseca T, Donald A, Bavetsias V, et al. (2006) Structure-based design of isoquinoline-5-sulfonamide inhibitors of protein kinase B. Bioorg Med Chem 14: 1255-1273.

5. Rouse MB, Seefeld MA, Leber JD, McNulty KC, Sun L, et al. (2009) Aminofurazans as potent inhibitors of AKT kinase. Bioorg Med Chem Lett 19: 1508-1511.
