# Supplementary material for: Knowledge-based Fragment Binding Prediction
Source: PLoS Comput Biol. 2014 Apr 24;10(4):e1003589. doi: 10.1371/journal.pcbi.1003589 (PMC3998881; doi:10.1371/journal.pcbi.1003589)
Supplement: Text S7 — Expanding the knowledge base beyond the information in the PDB. (DOCX) [file pcbi.1003589.s034.docx]

**Text S7. Expanding the knowledge base beyond the information in the PDB**

There are various approaches to extend the knowledge base of protein-fragment associations beyond those observed in the PDB. First, we can transfer fragment lists between microenvironments from homologous protein residues. This would enable usage of the many ligand-free structures that are homologous to ligand-bound structures.

Second, we can generalize the fragments linked to a microenvironment. A fragment can be generalized to fragments of high similarity, fragments from the same chemical motif (e.g. cyclic diols), or fragments from bioisosteric replacements (e.g. Cl, Br, SH, OH) [[1](#_ENREF_1)]. In the exotoxin A example, we observed nearest neighbor microenvironments to bind benzamide and fluorobenzamide fragments (Table S3). These fragments are bound by similar microenvironments and thus FragFEATURE when analyzing exotoxin A, retrieves both. As replacement of hydrogen with fluorine is a classical bioisosteric replacement, benzamide and fluorobenzamide could be generalized into a single entity. A possible information source for fragment generalization is SwissBioisostere [[2](#_ENREF_2)], which contains the performance of molecular replacements in biochemical assays.

Furthermore, only a subset of binding information from the ChEMBL database [[3](#_ENREF_3)] is present in the PDB in the form of a protein-small molecule complex. Realizing this information into 3D data such as by docking these compounds into their respective targets would increase the scope of the knowledge base, enabling microenvironment-fragment associations not directly observed in the PDB. As docking algorithms have limited accuracy, this information would be used as a supplementary source, down-weighted against the more reliable data that is extracted from the PDB. Increasing the number and scope of protein-fragment associations in the knowledge base may improve the predictive capabilities of FragFEATURE.

**References**

1. Meanwell NA (2011) Synopsis of some recent tactical application of bioisosteres in drug design. J Med Chem 54: 2529-2591.

2. Wirth M, Zoete V, Michielin O, Sauer WH (2013) SwissBioisostere: a database of molecular replacements for ligand design. Nucleic Acids Res 41: D1137-1143.

3. Gaulton A, Bellis LJ, Bento AP, Chambers J, Davies M, et al. (2012) ChEMBL: a large-scale bioactivity database for drug discovery. Nucleic Acids Res 40: D1100-1107.
