# Supplementary material for: Knowledge-based Fragment Binding Prediction
Source: PLoS Comput Biol. 2014 Apr 24;10(4):e1003589. doi: 10.1371/journal.pcbi.1003589 (PMC3998881; doi:10.1371/journal.pcbi.1003589)
Supplement: Text S8 — FragFEATURE and ligand/fragment affinity. (DOCX) [file pcbi.1003589.s035.docx]

**Text S8. FragFEATURE and ligand/fragment affinity**

There are assumptions and limitations associated with fragmenting PDB ligands as the source of fragment information. In building the knowledge base, we do not filter for ligand affinity as such information is not always available and thus imposing a minimal affinity requirement would reduce the scope of the knowledge base. We therefore assume the protein-ligand complexes from the PDB represent energetically favorable interactions. If indeed a complex possesses an unfavorable protein-fragment interaction (i.e. a fragment is bound due to favorable interactions from the rest of the ligand), we assume the interaction is unlikely to occur repeatedly in the PDB. As FragFEATURE predicts fragments that are statistically preferred by a set of query microenvironments, unfavorable protein-fragment interactions are not likely to achieve statistical significance. Statistical significance thus indicates repeated observation of the predicted fragment being bound by knowledge base microenvironments similar to the query, suggesting complementarity between the fragment and the microenvironments. Statistical significance, however, does not indicate strong fragment affinity, as we have neither filtered for high affinity ligands nor assigned fragments an affinity based on ligand affinity. FragFEATURE fragment predictions are likely to bind the pocket analyzed but require follow-up computational or experimental studies to determine the strength of the interaction.
