# Supplementary material for: Knowledge-based Fragment Binding Prediction
Source: PLoS Comput Biol. 2014 Apr 24;10(4):e1003589. doi: 10.1371/journal.pcbi.1003589 (PMC3998881; doi:10.1371/journal.pcbi.1003589)
Supplement: Text S9 — Microenvironment independence assumption. (DOCX) [file pcbi.1003589.s036.docx]

**Text S9. Microenvironment independence assumption**

FragFEATURE assumes independence of spatially proximal microenvironments in using Fisher’s method to combine fragment hypergeometric p-values. We believe this is a valid assumption because proximal microenvironments are generally of different types, such as the beta carbon of alanine and backbone nitrogen of alanine. These microenvironments are each compared to microenvironments of their respective type, preventing the two from retrieving the same nearest neighbors. Additionally, calculation of the microenvironments involved segmenting the local environment around a point of interest into a series of shells. Small changes in the central position cause protein atoms to shift into a different shell and thereby change the vector properties. Lastly, in unpublished work on calcium binding sites, we observed microenvironments shifted 0.5Å from a calcium binding position abolished signal. FEATURE microenvironments are thus sensitive to small shifts in the position of the microenvironment center suggesting microenvironment independence is a reasonable assumption.
