# Supplementary material for: Knowledge-based Fragment Binding Prediction
Source: PLoS Comput Biol. 2014 Apr 24;10(4):e1003589. doi: 10.1371/journal.pcbi.1003589 (PMC3998881; doi:10.1371/journal.pcbi.1003589)
Supplement: Text S10 — Multiple hypothesis correction. (DOCX) [file pcbi.1003589.s037.docx]

**Text S10. Multiple hypothesis correction**

For each microenvironment set, FragFEATURE tests multiple fragments for statistical significance but does not enforce a multiple hypothesis testing correction. A key concern is the non-independent nature of fragments. We fragment PDB ligands into overlapping fragments meaning testing a fragment is hand in hand with testing the substructures of the fragment. For example, testing a C_6_ fragment (six linear carbons) always occurs with testing of C_5_, C_4_, and C_3_. Such fragments are clearly correlated. Furthermore, the number of fragments tested is dependent on the type of fragment the microenvironment set prefers. Preference for an aromatic fragment involves fewer tests because aromatic rings are preserved during ligand fragmentation. In contrast, preference for a non-aromatic fragment involves many tests because there are more ways to fragment them. Multiple hypothesis testing correction is therefore not suitable because of inherent fragment correlation and biased penalty of non-aromatic fragments.

FragFEATURE also does not perform multiple hypothesis testing correction when testing multiple microenvironment sets for statistically significant fragments. Microenvironment sets are also highly correlated because they arise from generating exhaustive overlapping combinations of all spatially proximal microenvironments. Construction of the microenvironment sets is also not random but based on spatial information. With built in correlation between microenvironment sets as well as non-random set construction, multiple hypothesis testing correction did not appear essential.

More importantly, correction does not change the rank order of fragments. It mimics increasing the stringency of the significance threshold and thus increases method precision at the cost of recall. FragFEATURE’s performance on the validation ligands showed reasonable precision and no need for stricter significance thresholds. Furthermore, in conditions where all fragment predictions have weak p-values, a user may accept non-significant fragments in order to explore all interesting hypotheses.
